# A head-to-head comparison of the accuracy of commercially available large language models for infection prevention and control inquiries, 2024

**DOI:** 10.1017/ice.2024.205

**Published:** 2025-03

**Authors:** Oluchi J Abosi, Takaaki Kobayashi, Natalie Ross, Alexandra Trannel, Guillermo Rodriguez Nava, Jorge L. Salinas, Karen Brust

**Affiliations:** 1 University of Iowa Health Care, Iowa City, IA, USA; 2 Stanford University, Stanford, CA, USA

## Abstract

We investigated the accuracy and completeness of four large language model (LLM) artificial intelligence tools. Most LLMs provided acceptable answers to commonly asked infection prevention questions (accuracy 98.9%, completeness 94.6%). The use of LLMs to supplement infection prevention consults should be further explored.

## Background

Infection prevention and control (IPC) programs are essential in preventing healthcare-associated infections (HAIs) and ensuring patient and staff safety. They oversee HAI surveillance, outbreak investigation, policy development, provide guidance on infection transmission prevention, and occupational health.^1^ Their importance was emphasized during the COVID-19 pandemic, with a 500% surge in consultations.^2^

Artificial intelligence (AI) is a growing field in computer science that includes large language models (LLMs). LLMs are neural networks based on transformers simulating human responses.^3^ They have demonstrated capabilities in solving complex cases, excel in clinical reasoning, history-taking, and empathetic communication.^4^ For the lone infection preventionist (IP) or facilities without IP support, LLMs present the possibility for personalized support related to infection prevention inquiries. AI research in medicine primarily targets clinical contexts^4,5^, we investigated the accuracy and completeness of four LLMs using real-world infection prevention questions.

## Methods

The IP team at the University of Iowa Health Care Medical Center is led by hospital epidemiologists. It is an 866-bed tertiary care center encompassing 250 specialty and subspecialty outpatient clinics. IPs respond to urgent infection prevention questions via multiple communication modalities 24 hours a day. Domains covered include communicable infections, isolation precautions, environmental cleaning, public health inquiries, and laboratory testing. All calls received by the IP are recorded in a shared excel spreadsheet with date, time, and query details for each encounter.^2^

Using 2022 data, 31 sample questions were categorized into the three most common domains: transmission-based precautions, communicable disease exposures, and environmental cleaning (Supplemental Tables 1, 2). The study was approved as non-human subjects research by the University of Iowa Institutional Review Board.

We evaluated four LLMs: Microsoft Copilot (formerly Bing AI), GPT-3.5, GPT-4, and OpenEvidence between December 2023 and January 2024. These four LLMs were chosen for ease of use, access, and familiarity. Each LLM was queried once for each question. Two epidemiologists and one certified IP reviewed each question using 5-point and 6-point Likert scales for accuracy and completeness per our internal policies (Supplemental Table 3). Responses ≥3 were deemed accurate, and those ≥4 complete. We calculated acceptable accuracy percentages by dividing the number of responses with a score ≥3 by the total number of responses. Additional sensitivity analysis with accuracy scores of ≥ 4 was performed. Similarly, we calculated acceptable completeness percentages as the number of responses with a score ≥4 divided by the total number of responses. Each reviewer assessed 31 questions per LLM for both accuracy and completeness without a qualifying prompt. Additionally, we re-evaluated the quality of responses of the same 31 questions, restricting the LLMs to Centers for Disease Control and Prevention (CDC) databases by adding the statement: “Follow CDC guidelines in the United States.” We compared the accuracy and completeness of the LLMs using paired 2-tailed t-tests with Microsoft Copilot as the reference and calculated 95% confidence intervals from binomial distributions. Statistical analyses were performed using Python 3.10.

## Results

**Figure 1.**
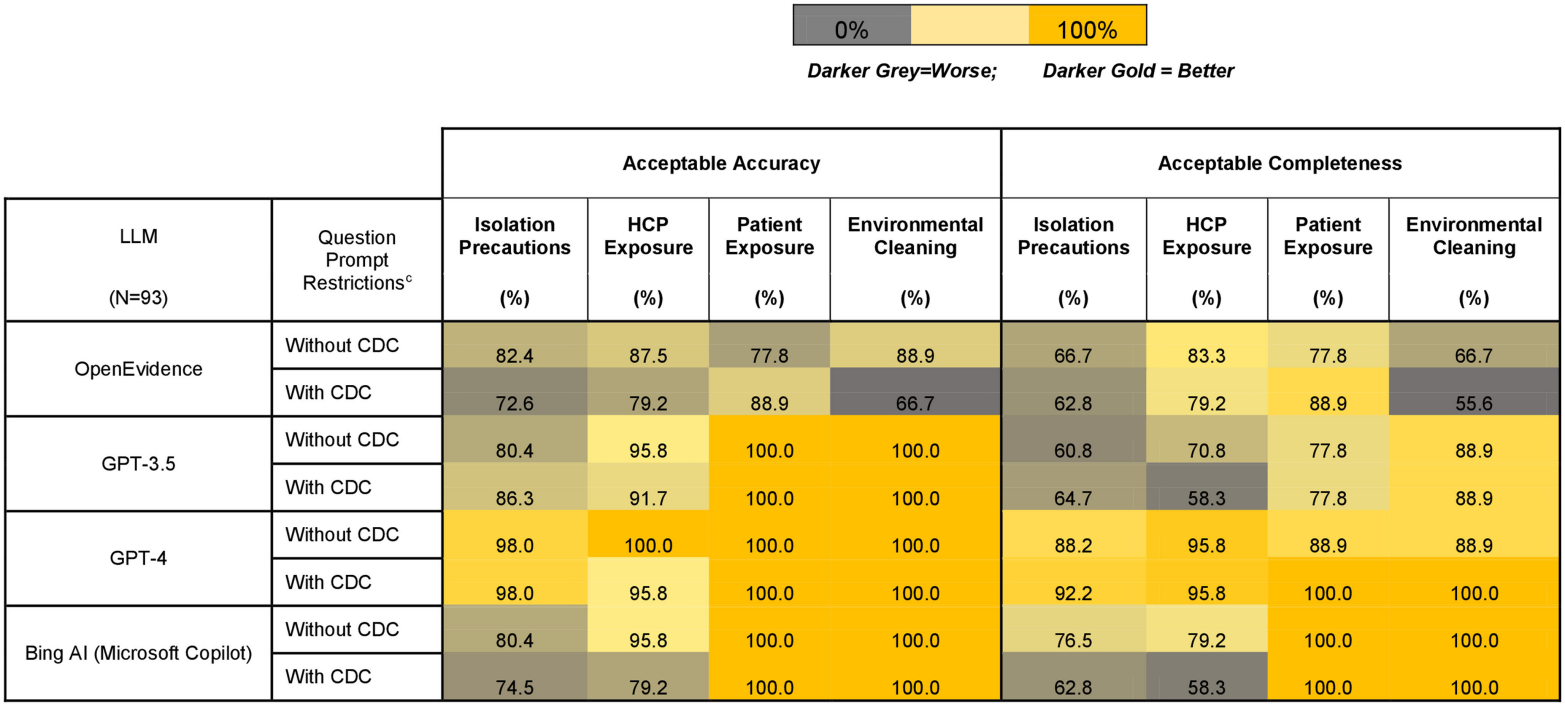
Heatmap of acceptable accuracy and completeness score percentages by category across large language models in response to infection prevention questions without and with CDC statements ^a, b^. LLM, Large Language Models. ^a^ The accuracy scale was a 5-point Likert scale (with 1 indicating completely incorrect; 2, more incorrect than correct; 3, More correct than incorrect but missing some major elements; 4, More correct than incorrect but missing some minor elements; and 5, completely correct). ^a^ The completeness scale was a 6-point Likert scale (1, addresses no aspect of the question, and the answer is not within the topic queried; 2, addresses no aspect of the question, and the answer is within the topic queried; 3, addresses some aspect of the question, but significant parts are missing or incomplete; 4, addresses most aspects of the questions but missing small details; 5, addresses all aspects of the question without additional information; and 6 addresses all aspects of the question and provides additional information beyond what was expected). ^b^ Responses with scores ≥3 were deemed accurate. Responses with scores ≥4 were deemed complete. ^c^Without limiting AI tool search to CDC-only references versus with prompt limiting AI tool search to CDC-only references.

Overall, GPT-4 had the highest accuracy: 98.9% (95% CI 94.3%–99.9%) without CDC restrictions and 97.9% (95% CI 92.6%%–99.6%) with restrictions (*P* < .001 for GPT-4 vs other LLMs) (Table 1). For specific domains, GPT-4 had the highest accuracy for isolation precaution responses (98.0% without CDC restrictions and 98.0% with restrictions, *P* < .001) and for healthcare personnel exposure responses (100% without CDC restrictions and 95.8% with restrictions, *P* < .001). OpenEvidence was the only LLM not reaching 100% accuracy for patient exposure and environmental cleaning responses (Figure 1, Supplemental Table 4).

**Table 1.** Percentage of overall acceptable accuracy and completeness score across large language models response to infection prevention questions without and with CDC statements^a,^ ^b^

	Acceptable Accuracy				Acceptable Completeness			
LLM	Without CDC statement^c^ (n = 93)		With CDC statement^c^ (n = 93)		Without CDC statement^c^ (n = 93)		With CDC statement^c^ (n = 93)	
	% (95% CI)	*P* value^d^	% (95% CI)	*P* value^d^	% (95% CI)	*P* value^d^	% (95% CI)	*P* value^d^
OpenEvidence	83.9 (74.8–90.4)	<0.001	75.3 (66.7–84.0)	<0.001	72.0 (61.9–80.3)	<0.001	68.8 (58.6–77.5)	0.16
GPT-3.5	88.2 (79.7–93.7)	0.76	90.3 (82.4–95.1)	<0.001	67.7 (57.6–76.6)	<0.001	66.7 (56.5–75.9)	<0.001
GPT-4	98.9 (94.3–99.9)	<0.001	97.9 (92.6–99.6)	<0.001	90.3 (82.4–95.1)	<0.001	94.6 (87.8–97.9)	<0.001
Bing AI (Microsoft Copilot)	88.2 (79.7–93.7)	Reference	80.7 (71.1–87.8)	Reference	81.7 (72.7–88.5)	Reference	68.8 (58.6–77.5)	Reference

LLMs, Large Language Models.

a The accuracy scale was a 5-point Likert scale (with 1 indicating completely incorrect; 2, more incorrect than correct; 3, approximately equal correct and incorrect; 4, more correct that incorrect; and 5, completely correct). The completeness scale was a 6-point Likert scale (with 1 indicating addresses no aspect of the question, and the answer is not within the topic queried; 2, addresses no aspect of the question, and the answer is within the topic queried; 3, addresses some aspect of the question, but significant parts are missing or incomplete; 4, addresses most aspects of the questions but missing small details; and 5, addresses all aspects of the question without additional information; 6 addresses all aspects of the question and provides additional information beyond what was expected).

b Responses with scores ≥3 was deemed accurate

c Without limiting the models search to CDC-only references versus with prompt limiting the models search to CDC-only references.

d Pairwise t-test results, with Bing AI (Microsoft Copilot) as reference

For completeness, GPT-4 led with 90.3% (95% CI 82.4%–95.1%) without CDC restrictions and 94.6% (95% CI 87.8%–97.9%) with restrictions (*P* < .001) (Table 1). Similarly, for specific domains, GPT-4 had the highest completeness for isolation precaution responses (88.2% without CDC restrictions and 92.2% with restrictions, p <.001) and for healthcare personnel exposure responses (95.8% with and without CDC restrictions, *P* < .001). Microsoft Copilot was the only LLM achieving 100% completeness for patient exposure responses, both with and without CDC restrictions (*P* < .05, except GPT-4 with CDC restrictions), and for environmental cleaning responses (*P* < .05, except GPT-4 with CDC restrictions) (Figure 1, Supplemental Table 5). The results of the sensitivity analysis are summarized in Supplemental Table 6.

GPT-3.5 and OpenEvidence lagged in both accuracy and completeness. Limiting LLMs to CDC-only references decreased response accuracy and completeness, particularly accuracy. Only GPT-3.5 and OpenEvidence showed improved accuracy with CDC restrictions for isolation precautions and patient exposures, respectively. With CDC restrictions, GPT-4 demonstrated improved completeness for isolation precautions, patient exposures, and environmental cleaning and disinfection. GPT-3.5 and OpenEvidence again showed improved completeness for isolation precautions and patient exposures, respectively.

## Discussion

Few studies have evaluated LLMs in practical IPC settings. A recent study evaluated the accuracy of AI tools in identifying HAIs concordant with National Healthcare Safety Network (NHSN) definitions using fictional scenarios. The AI tools correctly identified whether cases met NHSN definitions when given clear prompts.^6^ Herein, we evaluated the accuracy and completeness of four LLMs using real infection prevention queries. Most LLMs provided acceptable answers to the commonly asked inquiries. Prospective studies are needed to explore the application of AI in real-world IPC scenarios and assess the effectiveness of LLMs in infection control.

In this study, GPT-4 provided the most accurate and complete responses across most categories, whereas OpenEvidence was the least accurate and GPT-3.5 the least complete. LLMs performed better in areas concerning patient exposures and environmental cleaning, but their performance decreased in transmission-based precautions. Using CDC prompts negatively impacted accuracy and completeness, a trend consistent across all categories. However, using this prompt may not have been the best approach. OpenEvidence, which primarily searches PubMed or peer-reviewed articles, performed poorly, likely due to its limited references. Microsoft Copilot and both versions of ChatGPT have “knowledge” extracted from websites, textbooks, journals, and public-facing data sources. LLMs have the potential for continuous learning and improvement through regular exposure to new data and scenarios. By processing real-world infection prevention queries, these models can be continuously refined. Their accuracy and completeness can be improved by using techniques such as prompt engineering and retrieval-augmented generation without the need for extensive retraining, which requires significant computing power not readily available.^7^

After the COVID-19 pandemic, infection prevention consultation calls increased significantly. Although calls were primarily associated with COVID-19, numbers have not yet returned to pre-pandemic levels.^2^ The shift of focus from other infection prevention activities due to increased calls could have negative consequences and should be addressed.^8^ Integration of LLMs into IPC programs could significantly enhance resource allocation.^9^ LLMs efficiently handling frequently asked queries would allow human experts to concentrate on more complex and nuanced issues requiring specialized knowledge and critical thinking. Optimizing human resources would lead to more effective IPC programs and generate better overall healthcare outcomes.

Limitations include the small sample size of questions and evaluators from a single tertiary care center. We chose four readily available LLMs. Alternate LLMs or newer versions may provide different answers and impact results. Each LLM was queried only once. There is a lack of standardized methods to evaluate AI-generated responses. Although acceptable accuracy was scored at ≥3, we assigned this score for neutral categorization. A score of ≥4 would have resulted in fewer responses considered accurate (Supplemental Table 6). The magnitude of reduction was greater for LLMs with limited or outdated information sources. Additionally, not all LLMs provided output that included references or citations. However, we sought to be comprehensive by modifying assessment tools used in prior studies that assessed AI performance.^10^ Four categories were assessed, but each category had a different number of questions, ranging from 3 to 17, comparative to calls received. Additionally, although responses were independently reviewed by each evaluator, they could see responses to prompts with and without CDC restrictions, potentially influencing scoring.

In conclusion, LLMs show promise as tools to potentially supplement IPC programs and reduce workload. Their performances could be further enhanced through retrieval-augmented generation using official guidelines or local policies. Further development will enable LLMs to significantly contribute to the advancement of infection prevention practices.

## Supporting information

Abosi et al. supplementary material Abosi et al. supplementary material

## Supplementary material

The supplementary material for this article can be found at https://doi.org/10.1017/ice.2024.205.
