# Supplementary material for: A head-to-head comparison of the accuracy of commercially available large language models for infection prevention and control inquiries, 2024
Source: Infect Control Hosp Epidemiol. 2024 Dec 12;46(3):309–11. doi: 10.1017/ice.2024.205 (PMC11883648; doi:10.1017/ice.2024.205)
Supplement: Abosi et al. supplementary material [file S0899823X24002058sup001.docx]

**Supplemental Tables**

**Content**

**Table 1:** Questions Selected

**Table 2:** Question Categorization

**Table 3:** Rubric for Accuracy and Completeness Assessment

**Table 4:** Percentage of Acceptable Accuracy Score by Category Across Large Language Models Response to Infection Prevention Questions without and with CDC statements

**Table 5:** Percentage of Acceptable Completeness Score by Category Across Large Language Models Response to Infection Prevention Questions without and with CDC statements

**Table 6:** Sensitivity Analysis—Percentage of Overall Acceptable Accuracy Score (≥ 3 versus ≥4) Across Large Language Models Response to Infection Prevention Questions without and with CDC statements

**Table 7:** Comparison of Proportion of Overall Acceptable Accuracy Responses for each Large Language Models to Infection Prevention Questions without and with CDC statements

**Table 7(i):** Comparison of Proportion of Overall Acceptable Accuracy Responses by Category for each Large Language Models Response to Infection Prevention Questions without and with CDC statements

**Table 8:** Comparison of Proportion of Overall Completeness Responses for each Large Language Models to Infection Prevention Questions without and with CDC statements

**Table 8(i):** Comparison of Proportion of Overall Acceptable Completeness Responses by Category for each Large Language Models Response to Infection Prevention Questions without and with CDC statements

**Figure 1:** Heatmap of Acceptable Accuracy and Completeness Score by Category Across Large Language Models Response to Infection Prevention Questions without and with CDC statements

**Table 9:** Side-by-Side Questions and AI-Generated Responses without and with CDC Statement: All Categories, Selected Examples

**Table 1: Questions Selected.**

|  | Questions: |
| --- | --- |
| 1 | My patient tested positive for COVID-19. Do they need isolation? |
| 2 | If my patient tested positive for COVID-19 on October 23, 2023, when can they come out of isolation? They are immunocompetent. They are not in the ICU. |
| 3 | My patient tested positive for VRE. Do they need isolation? |
| 4 | My patient tested positive for rhinovirus. Do they need isolation? |
| 5 | My patient tested positive for influenza. Do they need isolation? |
| 6 | My patient tested positive for multi-drug resistant organism (MDRO). Do they need isolation? |
| 7 | My patient tested positive for shingles. Do they need isolation? |
| 8 | My patient tested positive for varicella zoster virus (VZV), in two dermatomes, that are contiguous. My patient is immunocompetent. Do they need isolation? |
| 9 | My patient tested positive for varicella zoster virus (VZV), in three dermatomes, that are not contiguous. My patient is immunosuppressed. Do they need isolation? |
| 10 | My patient tested positive for varicella zoster virus (VZV), in three dermatomes, that are not contiguous. Do I need to get tested for VZV? |
| 11 | My patient tested positive for varicella zoster virus (VZV), in three dermatomes, that are not contiguous. I was the nurse for one shift and I did not use personal protective equipment. I am pregnant. Do I need to get prophylaxis for VZV? |
| 12 | My patient tested positive for Bordetella pertussis (whooping cough). Do I need prophylaxis? |
| 13 | My patient tested positive for Neisseria meningitides (meningococcus). Do I need prophylaxis? |
| 14 | My patient's roommate tested positive for candida auris. What do I need to do for my patient? |
| 15 | My patient tested positive for TB by interferon gamma release assay. Do they need isolation? |
| 16 | My patient tested positive for TB by interferon gamma release assay. Patient's chest x-ray is normal. Do they need isolation? |
| 17 | My patient tested positive for c. diff by PCR alone. Do they need to be in isolation? |
| 18 | My patient tested positive for c diff by positive PCR and negative by antigen. Do they need to be in isolation? |
| 19 | My patient is in contact isolation for c diff. When can they come out of isolation? |
| 20 | My patient has lice. Do they need to be in isolation? |
| 21 | My patient has lice. They have received appropriate treatment. Do they still need to be in isolation? |
| 22 | My patient had a roommate that tested positive for COVID-19. What do I need to do for my patient? |
| 23 | My patient was diagnosed with pulmonary TB. I was the nurse for one shift. I did not use an N-95. Do I need to get tested for TB? |
| 24 | My patient came to clinic and was found to have bed bugs. What do we need to do with the clinic room? |
| 25 | My patient came to clinic and was found to have lice. What do we need to do with the clinic room? |
| 26 | My patient has pulmonary TB and is coming to an appointment tomorrow. What precautions should we take? |
| 27 | My patient came to clinic and was found to have COVID-19. There was an aerosol-generating procedure performed in the room. What do we need to do with the clinic room? |
| 28 | I stuck myself with a needle on a patient known to have HIV. Do I need HIV prophylaxis? |
| 29 | I stuck myself with a needle on a patient and we do not know the patient's HIV status. Do I need HIV prophylaxis? |
| 30 | I am a nurse and was bitten by a patient. The bite drew blood. Do I need to be tested for HIV and hepatitis? |
| 31 | I saw a bat in a patient's room in the neonatal intensive care unit. I did not come into contact with the bat. I called animal control, and they removed it. Does the patient need rabies prophylaxis? |

**Table 2: Question Categorization**

| **Question category** | **Question Numbers** |
| --- | --- |
| Isolation Precautions | 1,2,3,4,5,6,7,8,9,15,16,17,18,19,20,21,26 |
| Healthcare Personnel Exposure | 10,11,12,13,23,28,29,30 |
| Patient Exposure | 14,22,31 |
| Environmental Cleaning and Disinfection | 24,25,27 |

**Table 3: Rubric for Accuracy and Completeness Assessment**

| **Accuracy**​ | | |
| --- | --- | --- |
| **1**​ | Completely incorrect​ | **Not acceptable**​ |
| **2**​ | More incorrect than correct​ |  |
| **3**​ | More correct than incorrect but missing some major elements | **Acceptable**​ |
| **4**​ | More correct than incorrect but missing some minor elements |  |
| **5**​ | Completely correct​ |  |

| **Completeness**​ | | |
| --- | --- | --- |
| **1**​ | Addresses no aspect of the question, and the answer is not within the topic queried​ | **Not Acceptable**​ |
| **2**​ | Addresses no aspects of the question, but the answer is within the topic queried​ |  |
| **3**​ | Addresses some aspects of the question, but significant parts are missing or incomplete​ |  |
| **4**​ | Addresses most aspects of the question but missing small details​ | **Acceptable**​ |
| **5**​ | Addresses all aspects of the question without additional information ​ |  |
| **6**​ | Addresses all aspects of the question and provides additional information beyond what was expected​ |  |

**Table 4: Percentage of Acceptable Accuracy Score by Category Across Large Language Models Response to Infection Prevention Questions without and with CDC statements^a,b^**

| LLM | | Without CDC statement^c^  (N=93) | | With CDC statement^c^  (N=93) | |
| --- | --- | --- | --- | --- | --- |
|  |  | % (95% CI) | P value^d^ | % (95% CI) | P value^d^ |
| Isolation Precautions (n=51) | |  |  |  |  |
|  | OpenEvidence | 82.4 (69.7-90.7) | 0.82 | 72.6 (58.9-83.6) | <0.001 |
|  | GPT-3.5 | 80.4 (66.8-89.6) | 0.34 | 86.3 (73.7-93.4) | <0.001 |
|  | GPT-4 | 98.0 (89.6-99.9) | <0.001 | 98.0 (89.6-99.9) | <0.001 |
|  | Bing AI (Microsoft Copilot) | 80.4 (66.8-89.6) | Reference | 74.5 (60.9-84.8) | Reference |
| HCP Exposure (n=24) | |  |  |  |  |
|  | OpenEvidence | 87.5 (69.0-96.5) | <0.001 | 79.2 (58.5-91.4) | 0.96 |
|  | GPT-3.5 | 95.8 (79.6-99.8) | <0.001 | 91.7 (73.4-98.5) | <0.001 |
|  | GPT-4 | 100.0 (86.1-100.0) | <0.001 | 95.8 (79.6-99.8) | <0.001 |
|  | Bing AI (Microsoft Copilot) | 95.8 (79.6-99.8) | Reference | 79.2 (58.5-91.4) | Reference |
| Patient Exposure (n=9) | |  |  |  |  |
|  | OpenEvidence | 77.8 (44.2-95.9) | <0.001 | 88.9 (55.7-99.4) | <0.001 |
|  | GPT-3.5 | 100.0 (67.7-100.0) | 0.28 | 100.0 (67.7-100.0) | 0.28 |
|  | GPT-4 | 100.0 (67.7-100.0) | 0.42 | 100.0 (67.7-100.0) | 0.42 |
|  | Bing AI (Microsoft Copilot) | 100.0 (67.7-100.0) | Reference | 100.0 (67.67-100.0) | Reference |
| Environmental Cleaning and disinfection (n=9) | |  |  |  |  |
|  | OpenEvidence | 88.9 (55.7-99.4) | <0.001 | 66.7 (32.3-90.2) | <0.001 |
|  | GPT-3.5 | 100.0 (67.7-100.0) | 0.28 | 100.0 (67.7-100.0) | 0.28 |
|  | GPT-4 | 100.0 (67.7-100.0) | 0.42 | 100.0 (67.7-100.0) | 0.42 |
|  | Bing AI (Microsoft Copilot) | 100.0 (67.7-100.0) | Reference | 100.0 (67.7-100.0) | Reference |

LLMs, Large Language Models

^a^ The accuracy scale was a 5-point Likert scale (with 1 indicating completely incorrect; 2, more incorrect than correct; 3, More correct than incorrect but missing some major elements; 4, More correct than incorrect but missing some minor elements; and 5, completely correct)

^b^ Responses with scores ≥3 was deemed accurate

^c^Without limiting the models search to CDC only references versus with prompt limiting the models search to CDC only references.

**^d^** Pairwise t-test results, with Bing AI (Microsoft Copilot) as reference

**Table 5: Percentage of Acceptable Completeness Score by Category Across Large Language Models Response to Infection Prevention Questions without and with CDC statements^a,b^**

| LLM | | Without^b^ CDC statement  (N=93) | | With^b^ CDC statement  (N=93) | |
| --- | --- | --- | --- | --- | --- |
|  |  | % (95% CI) | P value^c^ | % (95% CI) | P value^c^ |
| Isolation Precautions(n=51) | |  |  |  |  |
|  | OpenEvidence | 66.7 (52.8-78.7) | <0.001 | 62.8 (48.7-75.3) | 0.05 |
|  | GPT-3.5 | 60.8 (46.9-73.7) | <0.001 | 64.7 (50.7-76.6) | 0.24 |
|  | GPT-4 | 88.2 (76.6-94.8) | <0.001 | 92.2 (81.6-97.3) | <0.001 |
|  | Bing AI (Microsoft Copilot) | 76.5 (62.8-86.6) | Reference | 62.8 (48.7-75.3) | Reference |
| HCP Exposure (n=24) | |  |  |  |  |
|  | OpenEvidence | 83.3 (62.8-94.1) | <0.001 | 79.2 (58.5-91.4) | <0.001 |
|  | GPT-3.5 | 70.8 (50.0-86.1) | <0.001 | 58.3 (37.2-76.6) | 0.63 |
|  | GPT-4 | 95.8 (79.6-99.8) | <0.001 | 95.8 (79.6-99.7) | <0.001 |
|  | Bing AI (Microsoft Copilot) | 79.2 (58.5-91.4) | Reference | 58.3 (37.2-76.6) | Reference |
| Patient Exposure (n=9) | |  |  |  |  |
|  | OpenEvidence | 77.8 (44.2-95.9) | <0.001 | 88.9 (55.7-99.4) | 0.001 |
|  | GPT-3.5 | 77.8 (44.2-95.9) | <0.001 | 77.9 (44.2-95.9) | <0.001 |
|  | GPT-4 | 88.9 (55.7-99.4) | <0.001 | 100.0 (67.7-100.0) | 0.43 |
|  | Bing AI (Microsoft Copilot) | 100.0 (67.7-100.0) | Reference | 100.0 (67.7-100.0) | Reference |
| Environmental Cleaning and disinfection(n=9) | |  |  |  |  |
|  | OpenEvidence | 66.7 (32.3-90.2) | <0.001 | 55.6 (24.1-83.1) | <0.001 |
|  | GPT-3.5 | 88.9 (55.7-99.4) | <0.001 | 88.9 (55.7-99.4) | <0.001 |
|  | GPT-4 | 88.9 (55.7-99.4) | <0.001 | 100.0 (67.7-100.0) | 0.43 |
|  | Bing AI (Microsoft Copilot) | 100.0 (67.7-100.0) | Reference | 100.0 (67.7-100.0) | Reference |

LLMs, Large Language Models

^a^ The completeness scale was a 6-point Likert scale (with 1 indicating addresses no aspect of the question, and the answer is not within the topic queried; 2, addresses no aspect of the question, and the answer is within the topic queried; 3, addresses some aspect of the question, but significant parts are missing or incomplete; 4, addresses most aspects of the questions but missing small details; and 5, addresses all aspects of the question without additional information; 6 addresses all aspects of the question and provides additional information beyond what was expected)

^b^ Without limiting the models search to CDC only references versus with prompt limiting the models search to CDC only references.

**^c^** Pairwise t-test results, with Bing AI (Microsoft Copilot) as reference

**Table 6: Sensitivity Analysis—Percentage of Overall Acceptable Accuracy Score (≥ 3 versus ≥4) Across Large Language Models Response to Infection Prevention Questions without and with CDC statements^a, b^**

| % Overall Acceptable Accuracy Across all LLM  (mean scores 3-5) | | | % Overall Acceptable Accuracy Across all LLM  (mean scores 4-5) | | |
| --- | --- | --- | --- | --- | --- |
| LLM | Without  CDC  (%)  (N=93) | With  CDC  (%)  (N=93) | LLM | Without  CDC  (%)  (N=93) | With  CDC  (%)  (N=93) |
| 1. OpenEvidence | 83.9 | 75.3 | 1. OpenEvidence | 66.7 | 66.7 |
| 2. ChatGPT-3.5 | 88.2 | 90.3 | 2. ChatGPT-3.5 | 65.6 | 66.7 |
| 3. ChatGPT-4.0 | 98.9 | 97.9 | 3. ChatGPT-4.0 | 85.0 | 86.0 |
| 4. Bing AI (Microsoft Copilot) | 88.2 | 80.7 | 4. Bing AI  (Microsoft Copilot) | 75.3 | 71.0 |

LLMs, Large Language Models

^a^ The accuracy scale was a 5-point Likert scale (with 1 indicating completely incorrect; 2, more incorrect than correct; 3, More correct than incorrect but missing some major elements; 4, More correct than incorrect but missing some minor elements; and 5, completely correct).

^b^ Responses deemed accurate with scores ≥3 compared to scores ≥4

^c^Without limiting the models search to CDC only references versus with prompt limiting the models search to CDC only references.

**^d^** Pairwise t-test results, with Bing AI (Microsoft Copilot) as reference

**Table 7: Comparison of Proportion of Overall Acceptable Accuracy Responses for each Large Language Models to Infection Prevention Questions without and with CDC statements^a, b^**

| LLM | Without CDC statement^c^  (N=93) | With CDC statement^c^  (N=93) | P value^d^ |
| --- | --- | --- | --- |
|  | % (95% CI) | % (95% CI) |  |
| OpenEvidence | 83.9 (74.8-90.4) | 75.3 (66.7-84.0) | <0.001 |
| GPT-3.5 | 88.2 (79.7-93.7) | 90.3 (82.4-95.1) | <0.001 |
| GPT-4 | 98.9 (94.3-99.9) | 97.9 (92.6-99.6) | 0.02 |
| Bing AI (Microsoft Copilot) | 88.2 (79.7-93.7) | 80.7 (71.1-87.8) | <0.001 |

LLM, Large Language Model

^a^ The accuracy scale was a 5-point Likert scale (with 1 indicating completely incorrect; 2, more incorrect than correct; 3, More correct than incorrect but missing some major elements; 4, , More correct than incorrect but missing some minor elements; and 5, completely correct)

^b^ Responses with scores ≥3 was deemed accurate

^c^Without limiting the models search to CDC only references versus with prompt limiting the models search to CDC only references.

**^d^** Pairwise t-test results, without CDC statement used as reference

**Table 7(i): Comparison of Proportion of Overall Acceptable Accuracy Responses by Category for each Large Language Models Response to Infection Prevention Questions without and with CDC statements^a,b^**

| LLM | | Without CDC statement^c^  (N=93) | With CDC statement^c^  (N=93) | | |
| --- | --- | --- | --- | --- | --- |
|  |  | % (95% CI) | % (95% CI) | P value^d^ | |
| Isolation Precautions (n=51) | |  |  |  | |
|  | OpenEvidence | 82.4 (69.7-90.7) | 72.6 (58.9-83.6) | <0.001 | |
|  | GPT-3.5 | 80.4 (66.8-89.6) | 86.3 (73.7-93.4) | <0.001 | |
|  | GPT-4 | 98.0 (89.6-99.9) | 98.0 (89.6-99.9) | 0.27 | |
|  | Bing AI (Microsoft Copilot) | 80.4 (66.8-89.6) | 74.5 (60.9-84.8) | <0.001 | |
| HCP Exposure (n=24) | |  |  |  | |
|  | OpenEvidence | 87.5 (69.0-96.5) | 79.2 (58.5-91.4) | <0.001 | |
|  | GPT-3.5 | 95.8 (79.6-99.8) | 91.7 (73.4-98.5) | <0.001 | |
|  | GPT-4 | 100.0 (86.1-100.0) | 95.8 (79.6-99.8) | <0.001 | |
|  | Bing AI (Microsoft Copilot) | 95.8 (79.6-99.8) | 79.2 (58.5-91.4) | <0.001 | |
| Patient Exposure (n=9) | |  |  | |  |
|  | OpenEvidence | 77.8 (44.2-95.9) | 88.9 (55.7-99.4) | 0.006 | |
|  | GPT-3.5 | 100.0 (67.7-100.0) | 100.0 (67.7-100.0) | 0.43 | |
|  | GPT-4 | 100.0 (67.7-100.0) | 100.0 (67.7-100.0) | 0.57 | |
|  | Bing AI (Microsoft Copilot) | 100.0 (67.7-100.0) | 100.0 (67.67-100.0) | 0.40 | |
| Environmental Cleaning and disinfection (n=9) | |  |  |  | |
|  | OpenEvidence | 88.9 (55.7-99.4) | 66.7 (32.3-90.2) | <0.001 | |
|  | GPT-3.5 | 100.0 (67.7-100.0) | 100.0 (67.7-100.0) | 0.43 | |
|  | GPT-4 | 100.0 (67.7-100.0) | 100.0 (67.7-100.0) | 0.57 | |
|  | Bing AI (Microsoft Copilot) | 100.0 (67.7-100.0) | 100.0 (67.7-100.0) | 0.40 | |

LLMs, Large Language Models

^a^ The accuracy scale was a 5-point Likert scale (with 1 indicating completely incorrect; 2, more incorrect than correct; 3, More correct than incorrect but missing some major elements; 4, More correct than incorrect but missing some minor elements; and 5, completely correct)

^b^ Responses with scores ≥3 was deemed accurate

^c^Without limiting the models search to CDC only references versus with prompt limiting the models search to CDC only references.

**^d^** Pairwise t-test results, without CDC statement used as reference

**Table 8: Comparison of Proportion of Overall Completeness Responses for each Large Language Models to Infection Prevention Questions without and with CDC statements^a, b^**

| LLM | Without CDC statement^c^  (N=93) | With CDC statement^c^  (N=93) | P value^d^ |
| --- | --- | --- | --- |
|  | % (95% CI) | % (95% CI) |  |
| OpenEvidence | 72.0 (61.9-80.3) | 68.8 (58.6-77.5) | <0.001 |
| GPT-3.5 | 67.7 (57.6-76.6) | 66.7 (56.5-75.9) | 0.41 |
| GPT-4 | 90.3 (82.4-95.1) | 94.6 (87.8-97.9) | <0.001 |
| Bing AI (Microsoft Copilot) | 81.7 (72.7-88.5) | 68.8 (58.6-77.5) | <0.001 |

LLMs, Large Language Models

^a^ The completeness scale was a 6-point Likert scale (with 1 indicating addresses no aspect of the question, and the answer is not within the topic queried; 2, addresses no aspect of the question, and the answer is within the topic queried; 3, addresses some aspect of the question, but significant parts are missing or incomplete; 4, addresses most aspects of the questions but missing small details; and 5, addresses all aspects of the question without additional information; 6 addresses all aspects of the question and provides additional information beyond what was expected)

^b^ Responses with scores ≥4 was deemed complete

^c^Without limiting the models search to CDC only references versus with prompt limiting the models search to CDC only references.

^d^Pairwise t-test results, without CDC statement used as reference.

**Table 8(i): Comparison of Proportion of Overall Acceptable Completeness Responses by Category for each Large Language Models Response to Infection Prevention Questions without and with CDC statements^a,b^**

| LLM | | Without^b^ CDC statement  (N=93) | With^b^ CDC statement  (N=93) | |
| --- | --- | --- | --- | --- |
|  |  | % (95% CI) | % (95% CI) | P value^c^ |
| Isolation Precautions(n=51) | |  |  |  |
|  | OpenEvidence | 66.7 (52.8-78.7) | 62.8 (48.7-75.3) | 0.001 |
|  | GPT-3.5 | 60.8 (46.9-73.7) | 64.7 (50.7-76.6) | <0.001 |
|  | GPT-4 | 88.2 (76.6-94.8) | 92.2 (81.6-97.3) | 0.003 |
|  | Bing AI (Microsoft Copilot) | 76.5 (62.8-86.6) | 62.8 (48.7-75.3) | <0.001 |
| HCP Exposure (n=24) | |  |  |  |
|  | OpenEvidence | 83.3 (62.8-94.1) | 79.2 (58.5-91.4) | 0.001 |
|  | GPT-3.5 | 70.8 (50.0-86.1) | 58.3 (37.2-76.6) | <0.001 |
|  | GPT-4 | 95.8 (79.6-99.8) | 95.8 (79.6-99.7) | 0.63 |
|  | Bing AI (Microsoft Copilot) | 79.2 (58.5-91.4) | 58.3 (37.2-76.6) | <0.001 |
| Patient Exposure (n=9) | |  |  |  |
|  | OpenEvidence | 77.8 (44.2-95.9) | 88.9 (55.7-99.4) | 0.006 |
|  | GPT-3.5 | 77.8 (44.2-95.9) | 77.9 (44.2-95.9) | 0.40 |
|  | GPT-4 | 88.9 (55.7-99.4) | 100.0 (67.7-100.0) | <0.001 |
|  | Bing AI (Microsoft Copilot) | 100.0 (67.7-100.0) | 100.0 (67.7-100.0) | 0.40 |
| Environmental Cleaning and disinfection(n=9) | |  |  |  |
|  | OpenEvidence | 66.7 (32.3-90.2) | 55.6 (24.1-83.1) | <0.001 |
|  | GPT-3.5 | 88.9 (55.7-99.4) | 88.9 (55.7-99.4) | 0.43 |
|  | GPT-4 | 88.9 (55.7-99.4) | 100.0 (67.7-100.0) | <0.001 |
|  | Bing AI (Microsoft Copilot) | 100.0 (67.7-100.0) | 100.0 (67.7-100.0) | 0.40 |

LLMs, Large Language Models

^a^ The completeness scale was a 6-point Likert scale (with 1 indicating addresses no aspect of the question, and the answer is not within the topic queried; 2, addresses no aspect of the question, and the answer is within the topic queried; 3, addresses some aspect of the question, but significant parts are missing or incomplete; 4, addresses most aspects of the questions but missing small details; and 5, addresses all aspects of the question without additional information; 6 addresses all aspects of the question and provides additional information beyond what was expected)

^b^ Without limiting the models search to CDC only references versus with prompt limiting the models search to CDC only references.

^c^Pairwise t-test results, without CDC statement used as reference.

**Figure 1: Heatmap of Acceptable Accuracy and Completeness Score Percentages by Category Across Large Language Models in Response to Infection Prevention Questions without and with CDC statements a, b**

Figure legend.

LLM, Large Language Models

a The accuracy scale was a 5-point Likert scale (with 1 indicating completely incorrect; 2, more incorrect than correct; 3, More correct than incorrect but missing some major elements; 4, More correct than incorrect but missing some minor elements; and 5, completely correct)

a The completeness scale was a 6-point Likert scale (1, addresses no aspect of the question, and the answer is not within the topic queried; 2, addresses no aspect of the question, and the answer is within the topic queried; 3, addresses some aspect of the question, but significant parts are missing or incomplete; 4, addresses most aspects of the questions but missing small details; 5, addresses all aspects of the question without additional information; and 6 addresses all aspects of the question and provides additional information beyond what was expected)

b Responses with scores ≥3 were deemed accurate. Responses with scores ≥4 were deemed complete.

cWithout limiting AI tool search to CDC only references versus with prompt limiting AI tool search to CDC only references.

**Table 9: Side by side Questions and AI-Generated Responses Without and With CDC Statement: All Categories, Selected Examples**

| Questions | AI- Generated Responses (without and with CDC Restrictions) | | | | | | | | | | | | | | | |
| --- | --- | --- | --- | --- | --- | --- | --- | --- | --- | --- | --- | --- | --- | --- | --- | --- |
|  | OpenEvidence | | | | ChatGPT 3.5 | | | | ChatGPT 4.0 | | | | Bing | | | |
| Isolation Precautions | | | | | | | | | | | | | | | | |
| My patient tested positive for COVID-19. Do they need isolation? | Yes, patients who test positive for COVID-19 need to be isolated. The European Society for Medical Oncology (ESMO) recommends that patients confirmed as COVID-19 positive should be admitted to segregated wards, and patients under investigation for COVID-19 should also be segregated until a negative swab is obtained.[1] The World Society of Emergency Surgery (WSES) suggests that after hospital discharge, all the confirmed asymptomatic surgical COVID-19 patients should be kept in isolation for at least 2 weeks have passed since their first positive RT-PCR nasopharyngeal swab test.[2] The Evidence-Based Medicine Chapter of China International Exchange and Promotive Association for Medical and Health Care (CPAM) and Chinese Research Hospital Association (CRHA) also suggest that strict self-isolation protocols and extended follow-up periods might be needed for recovered COVID-19 patients.[3] However, it's important to note that the duration of isolation may vary depending on the patient's symptoms and the results of subsequent testing. | | Yes, according to the United States Centers for Disease Control and Prevention (CDC), patients who test positive for COVID-19 are recommended to isolate. The CDC advises that individuals who had COVID-19 symptoms can discontinue isolation once it has been 10 days since symptom onset, they have been afebrile for at least 24 hours without the use of fever-reducing medications, and their other COVID-19–related symptoms (except loss of taste or smell) are improving.[1] However, it's important to note that some patients may require prolonged isolation beyond the standard 10 days, particularly those who are critically ill or immunocompromised.[2] | | I am not a medical professional, but I can provide some general information. It's crucial to consult with a healthcare professional for advice tailored to your specific situation. As of my last knowledge update in January 2022, guidelines may have changed, so please verify this information with up-to-date sources.  Typically, individuals who test positive for COVID-19 are advised to isolate themselves to prevent the spread of the virus to others. Isolation helps protect family members, friends, and the community from potential exposure. Here are some general recommendations: 1. **Home Isolation:** If the patient's symptoms are mild to moderate and they do not require hospitalization, they are often asked to isolate at home. This involves staying in a separate room, using a separate bathroom if possible, and avoiding contact with other household members. 2. **Duration of Isolation:** The duration of isolation may vary, but it is generally recommended until a certain number of days have passed since the onset of symptoms or the date of the positive test result. Guidelines may change, so it's important to follow the advice provided by healthcare authorities in your region. 3. **Follow Healthcare Provider Guidance:** Always follow the guidance provided by healthcare professionals or public health authorities. They will provide specific instructions based on the patient's condition, severity of symptoms, and any underlying health conditions. 4. **Monitoring Symptoms:** It's important to monitor the patient's symptoms and seek medical attention if their condition worsens. Emergency warning signs may include difficulty breathing, persistent chest pain or pressure, confusion, inability to stay awake, or bluish lips or face. 5. **Informing Close Contacts:** Inform close contacts about the positive test result so that they can take appropriate precautions. Close contacts may be advised to quarantine.  Remember, guidelines and recommendations may vary based on local health department guidelines and the patient's specific circumstances. Always consult with healthcare professionals for the most accurate and current information. | | Yes, according to the United States Centers for Disease Control and Prevention (CDC) recommendations, individuals who test positive for COVID-19 should isolate themselves to prevent the spread of the virus. Here are the general guidelines for isolation:  Stay at home: The individual should stay at home and avoid going to work, school, or any public places. Isolate from others: They should stay in a separate room and use a separate bathroom if possible. If not possible, the bathroom should be cleaned after each use. Wear a mask: When the person is around others or animals, they should wear a mask to reduce the risk of spreading the virus. Avoid close contact: They should avoid close contact with other household members and pets. Maintain a distance of at least 6 feet. Hand hygiene: Frequent handwashing with soap and water for at least 20 seconds is essential. Disinfect frequently touched surfaces: Regularly clean and disinfect surfaces that are touched often, such as doorknobs, light switches, and countertops. Follow healthcare provider's advice: It's important to follow the guidance provided by their healthcare provider, and they should notify their close contacts of their positive test result. The duration of isolation may vary depending on the severity of the illness and individual circumstances, so it's advisable to consult with a healthcare professional for specific guidance. It's crucial to follow CDC recommendations to help prevent the spread of COVID-19. | | If your patient has tested positive for COVID-19, they typically need to follow isolation guidelines to prevent the spread of the virus to others. The specific recommendations can vary depending on the public health guidelines in your region and the current understanding of the virus. As of my last update in April 2023, common recommendations for COVID-19 positive individuals included:  1. **Isolation Period**: Patients are usually advised to isolate for a certain period, often around 5-10 days, but this can vary. The duration of isolation can depend on the severity of symptoms and whether the person is immunocompromised. 2. **Symptom Monitoring**: The patient should monitor their symptoms. If symptoms worsen, they may need to seek medical attention. 3. **Avoiding Contact with Others**: During the isolation period, the patient should stay in a separate room and use a separate bathroom if possible. They should avoid contact with other household members and pets. 4. **Wearing Masks**: If they need to be around others or leave their isolation space, they should wear a mask. 5. **Ending Isolation**: Guidance on when to end isolation can depend on symptom improvement and sometimes a negative test result. However, it's important to consult the latest guidelines from health authorities such as the World Health Organization (WHO), Centers for Disease Control and Prevention (CDC), or your local health department, as recommendations can change with new data and the evolving situation. Always refer to the most current and locally relevant guidelines for the most accurate advice. | | The guidance from the United States Centers for Disease Control and Prevention (CDC) for managing a patient who tests positive for COVID-19 includes the following key points:  Isolation: Individuals who test positive for COVID-19 should isolate. This means staying away from others, including those in their household, as much as possible.  Duration of Isolation: The duration of isolation can vary based on symptoms and severity of the case. Generally, isolation can end after at least 5 days if they are asymptomatic or their symptoms are resolving (without fever for 24 hours, without the use of fever-reducing medications). A longer isolation period may be required for those with severe illness or weakened immune systems.  After Isolation: After ending isolation, it's recommended to continue wearing a mask around others for an additional 5 days.  Close Contacts: Close contacts of the infected individual should be informed and should follow CDC guidelines for exposure, which may include testing and self-monitoring for symptoms.  Medical Care: If the patient's symptoms worsen or become severe (such as difficulty breathing, persistent pain or pressure in the chest, new confusion, inability to wake or stay awake, or bluish lips or face), they should seek medical attention immediately.  It's important to stay updated with the latest CDC guidelines, as recommendations can change with new information about the virus. For the most current advice and detailed guidance, visiting the CDC's official website or consulting with a healthcare professional is advisable. | | I’m sorry to hear that your patient tested positive for COVID-19. According to the CDC, people who are infected but asymptomatic or people with mild COVID-19 should isolate through at least day 5 (day 0 is the day symptoms appeared or the date the specimen was collected for the positive test for people who are asymptomatic). They should wear a mask through day 10. A test-based strategy may be used to remove a mask sooner. People with moderate or severe COVID-19 should isolate through at least day 10. Those with severe COVID-19 may remain infectious beyond 10 days and may need to extend isolation for up to 20 days. People who are moderately or severely immunocompromised should isolate through at least day 20. Use of serial testing and consultation with an infectious disease specialist is recommended in these patients prior to ending isolation. | | Yes, according to the United States Centers for Disease Control and Prevention (CDC), a patient who tests positive for COVID-19 should isolate. Here are the key points from the CDC’s recommendations:  Isolation period: The patient should stay home and isolate from others in their home for at least 5 days. Day 0 is the day symptoms appeared or the date the specimen was collected for the positive test for people who are asymptomatic. Masking: The patient should wear a high-quality mask if they must be around others at home and in public. End of isolation: Isolation can end after at least 5 days if fever has resolved for at least 24 hours (without taking fever-reducing medications) and other symptoms are improving. However, the patient should continue to wear a mask around others through day 10. Please note that these guidelines are subject to change and it’s always best to check the latest recommendations from the CDC. Also, this information is intended for a general audience. Healthcare professionals should see specific guidelines for healthcare settings. This CDC guidance is meant to supplement—not replace—any federal, state, local, territorial, or tribal health and safety laws, rules, and regulations. If symptoms recur or worsen, the isolation period should restart.  Please ensure your patient follows these guidelines and consults with a healthcare professional for personalized advice. Stay safe! | |
|  | Accuracy: 4 | Completeness: 4 | Accuracy:5 | Completeness: 6 | Accuracy: 3 | Completeness: 3 | Accuracy:3 | Completeness: 3 | Accuracy: 4 | Completeness: 3 | Accuracy:5 | Completeness: 6 | Accuracy: 5 | Completeness: 6 | Accuracy:4 | Completeness: 5 |
| My patient tested positive for VRE. Do they need isolation? | The decision to isolate patients who test positive for Vancomycin-Resistant Enterococci (VRE) can depend on the specific genotype of the VRE. A study found that patients colonized with VRE of genotype vanC did not require contact isolation, as they were not associated with outbreaks or significant rates of bloodstream infection.[1] This suggests that not all patients with VRE need to be isolated, particularly if they are colonized with the vanC genotype. However, it's important to note that other genotypes, such as vanA or vanB, may still require isolation due to their potential for causing outbreaks and severe infections.[1] Risk factors for VRE colonization include previous antibiotic therapy, particularly with piperacillin/tazobactam, meropenem, and vancomycin, as well as previous dialysis.[2] Other risk factors include postoperative critical care, cardiovascular diseases, and the presence of an endotracheal tube.[3] In patients with malignancies and fever and neutropenia, the American Society of Clinical Oncology and the Infectious Diseases Society of America recommend considering modifications to initial empirical therapy for patients at risk for infection with antibiotic-resistant organisms, including VRE.[4] In conclusion, the need for isolation in patients who test positive for VRE may depend on the specific genotype of the VRE and the patient's individual risk factors. | | The United States Centers for Disease Control and Prevention (CDC) recommends contact precautions for patients who test positive for vancomycin-resistant enterococci (VRE). This includes the use of personal protective equipment (PPE) such as gloves and gowns, and the patient being placed in a private room or cohorting with other patients with the same infection. However, the need for isolation may depend on the specific circumstances. For example, in situations where MRSA and VRE are endemic, contact isolation precautions for visitors may not be necessary in routine circumstances.[1] However, visitors who will be interacting with multiple patients should use isolation practices similar to healthcare workers.[1] It's also important to note that not all patients with VRE may need to be isolated. For instance, carriers of VRE of genotype vanC may not require contact isolation.[2] However, discontinuing contact precautions for VRE has been associated with rising VRE bloodstream infection rates in some settings.[3] Therefore, the decision to isolate should be made on a case-by-case basis, considering factors such as the specific genotype of VRE, the prevalence of VRE in the community, and the risk of transmission to other patients and visitors. | | Vancomycin-resistant Enterococci (VRE) are bacteria that have developed resistance to the antibiotic vancomycin. In healthcare settings, the decision to isolate a patient with VRE depends on various factors, including the patient's overall health, the specific circumstances, and the hospital's infection control policies.  Isolation precautions are often implemented to prevent the spread of VRE to other patients and healthcare workers. Common precautions for VRE may include contact precautions, which involve using gloves and gowns when entering the patient's room to minimize direct contact with the patient and their surroundings.  It's important to follow the guidelines and recommendations provided by the infection control team at your healthcare facility. They will consider the specific details of the case, such as the patient's clinical condition, the type of care being provided, and the potential risk of transmission.  Always consult with your facility's infection control team or the healthcare provider responsible for the patient's care to determine the appropriate isolation measures based on the current protocols and guidelines in place. | | According to the United States Centers for Disease Control and Prevention (CDC) guidelines, patients who test positive for VRE (Vancomycin-resistant Enterococcus) may need isolation depending on their individual circumstances and the facility's infection control policies. The decision on isolation should be made in consultation with your facility's infection control team or local health authorities.  Isolation measures for patients with VRE may include:  Contact Precautions: Patients with VRE should be placed in a single-patient room, and healthcare workers should wear gloves and gowns when entering the room to provide care.  Hand Hygiene: Emphasize proper hand hygiene for both healthcare workers and patients to prevent the spread of VRE.  Environmental Cleaning: Ensure thorough cleaning and disinfection of surfaces in the patient's room and surrounding areas.  Education: Educate the patient and their family members about infection prevention measures and the importance of following the recommended precautions.  It's important to note that the specific isolation and infection control measures may vary depending on the patient's clinical condition, the type of healthcare facility, and local guidelines. Therefore, it is advisable to consult with your facility's infection control experts and follow their recommendations to prevent the spread of VRE and ensure the safety of both patients and healthcare workers. | | Vancomycin-resistant Enterococci (VRE) is a type of bacteria that is resistant to the antibiotic vancomycin, which is often used to treat serious infections. In healthcare settings, VRE can be spread from one person to another, particularly in hospitals or long-term care facilities.   Patients who test positive for VRE typically require isolation to prevent the spread of the bacteria to other patients, especially those who are immunocompromised or have open wounds. The specific isolation precautions can include:  1. **Contact Precautions:** This involves wearing gloves and gowns when caring for or visiting the patient.  2. **Private Room or Cohorting:** Ideally, the patient should be placed in a private room. If this is not possible, they may be placed in a room with another patient who has the same infection (cohorting). 3. **Hand Hygiene:** Hand washing is crucial. Healthcare providers, patients, and visitors should wash their hands thoroughly after contact with the patient or the patient's environment. 4. **Environmental Cleaning and Disinfection:** Regular cleaning and disinfection of surfaces and medical equipment in the patient's room is important.  The duration of these precautions can vary and is usually determined by the healthcare facility's infection control policy and the patient's clinical situation. It's essential to consult with the infection control team at your facility for specific guidelines. Remember, strict adherence to these precautions is key in preventing the spread of VRE. | | Yes, patients who have tested positive for Vancomycin-Resistant Enterococci (VRE) generally require isolation according to the guidelines of the United States Centers for Disease Control and Prevention (CDC). Isolation precautions are important to prevent the spread of VRE, particularly in healthcare settings.  The CDC recommends the following for managing patients with VRE: Contact Precautions: Patients should be placed under contact precautions. This means healthcare workers and visitors should use gloves and gowns when entering the patient's room. Private Room: Ideally, the patient should be placed in a private room. If a private room is not available, the patient can be placed in a room with another patient with the same infection (cohorting). Hand Hygiene: Frequent hand washing with soap and water or using an alcohol-based hand sanitizer is crucial for everyone entering or leaving the patient’s room. Environmental Cleaning and Disinfection: Regular and thorough cleaning and disinfection of the patient's environment and equipment are essential to prevent the spread of VRE. Patient Education: Educating the patient and their family about VRE, the importance of hand hygiene, and other precautions is vital for controlling the spread. Antibiotic Stewardship: Judicious use of antibiotics is important in controlling VRE. Antibiotic stewardship programs help in optimizing the treatment of infections and reducing the emergence of resistant bacteria. Monitoring and Surveillance: Ongoing surveillance of VRE cases is important to understand and control its spread within the healthcare setting.  Always follow your facility's specific protocols and guidelines in addition to the CDC recommendations. If you have specific questions about managing a VRE case, consult with your facility's infection control team or the CDC guidelines for the most current and detailed information. | | Yes, patients who test positive for Vancomycin-resistant enterococci (VRE) should be placed in isolation. The best practice is to place VRE-infected or colonized patients in private rooms or in the same room as other patients who have VRE. It’s also recommended to wear gloves (clean, nonsterile gloves are adequate) when entering the room of a VRE-infected or colonized patient because VRE can extensively contaminate such an environment. These measures are part of the guidelines for control of VRE spread recommended by health agencies like the Centers of Disease Control and Prevention. They include controlling VRE spread through vancomycin usage, screening and isolation of patients with VRE in hospital settings, education, cleaning, and contact precautions. | | Yes, according to the United States Centers for Disease Control and Prevention (CDC), patients who test positive for Vancomycin-resistant Enterococci (VRE) should be isolated to prevent patient-to-patient transmission. Here are some key recommendations:  Place VRE-infected or colonized patients in private rooms or in the same room as other patients who have VRE. Wear gloves when entering the room of a VRE-infected or colonized patient because VRE can extensively contaminate such an environment. Keep hands clean to avoid getting sick and spreading germs that can cause infections. Frequently clean areas of the home, such as bathrooms, that may become contaminated with VRE. Wear gloves if hands may come in contact with body fluids that may contain VRE, such as stool or bandages from infected wounds. Inform healthcare providers if you or someone you care for has VRE so that appropriate precautions can be taken to prevent spread. Please note that these are general guidelines and the specific measures may vary depending on the patient’s condition and the healthcare setting. It’s always best to consult with the healthcare team for the most appropriate course of action. | |
|  | Accuracy:4 | Completeness: 3 | Accuracy:5 | Completeness: 6 | Accuracy: 3 | Completeness: 3 | Accuracy:3 | Completeness: 4 | Accuracy: 3 | Completeness: 3 | Accuracy: 4 | Completeness: 4 | Accuracy: 2 | Completeness: 3 | Accuracy: 2 | Completeness: 3 |
| HCP Exposure | | | | | | | | | | | | | | | | |
| My patient tested positive for varicella zoster virus (VZV), in three dermatomes, that are not contiguous. I was the nurse for one shift and I did not use personal protective equipment. I am pregnant. Do I need to get prophylaxis for VZV? | In the case of exposure to varicella zoster virus (VZV) during pregnancy, prophylaxis is recommended to prevent maternal infection and subsequent complications. The British Society for the Study of Infection advises that varicella-zoster immune globulin (VZIG) should be administered as soon as possible, preferably within 96 hours from exposure.[1] This is particularly important as primary infection with VZV in the final weeks of pregnancy may cause transplacental infection and neonatal varicella, with infants at highest risk if born from 5 days before to 2 days after onset of the maternal varicella rash.[2] However, the effectiveness of VZIG for prophylaxis beyond 96 hours post-exposure has not been evaluated.[1] If maternal infection does occur, intravenous acyclovir is recommended for treatment, particularly in cases of maternal pneumonia or severe neonatal disease.[1] It's important to note that the risk of complications varies depending on the trimester of pregnancy during which exposure occurs. Infection during the first and second trimesters may increase the risk for congenital varicella syndrome, while third trimester infection may lead to maternal pneumonia, which can be life-threatening if not treated appropriately.[1] In summary, prophylaxis with VZIG is recommended following exposure to VZV in pregnancy, ideally within 96 hours of exposure. If infection occurs, treatment with acyclovir is advised. | | If a pregnant individual has been exposed to a patient who tested positive for varicella zoster virus (VZV), prophylaxis is recommended to prevent maternal infection and subsequent complications. According to the British Society for the Study of Infection, varicella-zoster immune globulin (VZIG) should be administered as soon as possible, preferably within 96 hours from exposure.[1] The effectiveness of VZIG beyond 96 hours has not been evaluated.[1] Varicella infection during pregnancy can lead to serious complications. In the first and second trimester, it may increase the risk for congenital varicella syndrome 0.5–1.5% above the baseline risk for major malformation.[1] In the third trimester, it may lead to maternal pneumonia, which can be life-threatening if not treated appropriately.[1] It's important to note that no controlled study has yet evaluated the effectiveness of acyclovir or valacyclovir for postexposure prophylaxis to pregnant women or neonates.[1] Therefore, the use of these antiviral medications for prophylaxis in this context is not currently supported by evidence. The International Society of Ultrasound in Obstetrics and Gynecology also provides guidance on the risk of congenital varicella syndrome, which is estimated to be 0.5% if maternal infection was in the first 13 gestational weeks, and 2% for infections between weeks 13 and 20.[2] The risk for congenital varicella syndrome is minimal after this point; however, there is a 25% risk of clinical neonatal varicella if infection occurs after 36 weeks.[2] In conclusion, prophylaxis with VZIG is recommended following exposure to VZV in pregnancy, ideally within 96 hours of exposure. | | I am not a healthcare professional, but I can provide you with some general information that might be helpful. It's crucial to consult with your healthcare provider for advice tailored to your specific situation.  Varicella zoster virus (VZV) causes chickenpox and can also lead to shingles later in life. If you have been exposed to a patient with a confirmed VZV infection and you are pregnant, it is important to discuss this with your healthcare provider as soon as possible. Pregnant women who are exposed to VZV and are not immune may be at risk for complications.  Your healthcare provider will consider factors such as your immune status, gestational age, and any previous history of chickenpox or vaccination. In some cases, post-exposure prophylaxis with varicella-zoster immune globulin (VZIG) may be recommended to reduce the risk of severe infection in pregnant women.  Again, it's crucial to consult with your healthcare provider promptly to discuss your specific situation and get appropriate guidance. They will be able to assess the risk and recommend any necessary interventions to ensure the health and safety of both you and your baby. | | I'm not a medical professional, but I can provide some general information. It's important to consult with a healthcare provider for personalized advice in your situation.  Varicella zoster virus (VZV) can cause chickenpox and shingles. If you have been in close contact with a patient who has tested positive for VZV and you are pregnant, it's essential to consult your healthcare provider immediately. Pregnant individuals can be at risk of complications if they contract VZV, especially during certain stages of pregnancy.  Your healthcare provider will consider various factors, including the timing of exposure, your vaccination history, and your pregnancy status, to determine if you need prophylaxis or any specific precautions. Following the recommendations of the United States Centers for Disease Control and Prevention (CDC) is important in such cases, but individualized medical advice is crucial for your specific situation.  Please reach out to your healthcare provider or a medical professional as soon as possible to discuss your situation and receive guidance on whether prophylaxis or other measures are necessary. | | If you have been exposed to Varicella Zoster Virus (VZV) and you're pregnant, it's crucial to take appropriate steps to ensure your safety and that of your unborn child. Here's what you should consider:  1. **Immunity Status**: If you've previously had chickenpox or received the varicella vaccine, you might already be immune. However, it's essential to confirm this with a blood test to check for varicella immunity.  2. **Risk of Exposure**: Being in close contact with a patient with VZV increases your risk, especially if you did not use personal protective equipment.   3. **Prophylaxis Options**: Varicella Zoster Immune Globulin (VZIG) or antiviral medications may be considered for prophylaxis. However, the decision to use these depends on several factors, including your immunity status and the timing of exposure.  4. **Consult Your Healthcare Provider**: It's vital to inform your healthcare provider about your exposure. They can assess your risk, conduct necessary tests, and recommend appropriate prophylaxis if needed.  5. **Timing**: If prophylaxis is required, it's generally most effective when administered within 96 hours of exposure.  6. **Monitoring for Symptoms**: Watch for any signs of VZV infection, such as rash or fever, and seek medical attention promptly if symptoms develop.  7. **Preventive Measures**: Practice good hand hygiene and avoid further exposure to VZV.  Remember, every situation is unique, and the best course of action depends on individual circumstances. Consulting with your healthcare provider is the most reliable way to ensure you receive the appropriate care and guidance. | | If you are a healthcare professional who has been exposed to varicella zoster virus (VZV) and are pregnant, it's important to follow specific guidelines to ensure your safety and the safety of your unborn child.  The CDC recommends the following steps for healthcare personnel exposed to VZV:  Determine Immunity: First, ascertain if you are immune to VZV. Immunity is typically confirmed if you have a history of varicella (chickenpox) or herpes zoster (shingles), documentation of two doses of varicella vaccine, or a positive VZV IgG antibody test.  Consult a Healthcare Provider: If you are not immune or unsure about your immunity status, consult a healthcare provider immediately. They may recommend varicella zoster immune globulin (VZIG) or other prophylactic treatments to reduce the risk of contracting the virus.  Monitor for Symptoms: Be vigilant for symptoms of varicella, such as fever, fatigue, and a characteristic rash, for up to 21 days after exposure (up to 28 days if you receive VZIG).  Follow-Up Care: Regular prenatal care and follow-up are crucial. If you develop any symptoms suggestive of varicella, contact your healthcare provider immediately.  Work Restrictions: The CDC may advise healthcare workers who are not immune to varicella to avoid exposure to high-risk patients until their immunity is confirmed or they have passed the incubation period without symptoms.  It's essential to follow the guidance of your healthcare provider and your workplace's infection control policies. They can offer personalized advice based on your immunity status, the nature of the exposure, and your health history. | | Hello, this is Bing. I’m sorry to hear that you were exposed to varicella zoster virus (VZV) in your workplace. VZV is the virus that causes chickenpox and shingles. It can be transmitted through contact with skin lesions or respiratory secretions of an infected person.  According to the CDC, pregnant women are at higher risk for severe disease and complications from VZV infection. Therefore, they should receive varicella zoster immune globulin (VariZIG) if they are at risk for severe disease and varicella vaccination is contraindicated. VariZIG is a medication that contains antibodies that can help prevent or reduce the severity of VZV infection.  The CDC also recommends that healthcare personnel who are exposed to VZV should receive postexposure prophylaxis (PEP) as soon as possible and within 10 days of first exposure. PEP consists of two doses of VariZIG given 24 hours apart, followed by a single dose of acyclovir 5 mg/kg given 48 hours after the second dose of VariZIG. PEP can help prevent or reduce the severity of VZV infection in healthcare personnel who are at risk for severe disease.  Since you were the nurse for one shift and you did not use personal protective equipment (PPE), you may have been exposed to VZV from your patient. You should contact your healthcare provider as soon as possible and follow their advice on whether you need to get PEP or not. You should also monitor your health for any signs or symptoms of VZV infection, such as fever, rash, headache, muscle aches, or nerve pain. If you develop any of these symptoms, you should seek medical attention immediately. | | According to the United States Centers for Disease Control and Prevention (CDC), if a pregnant woman is exposed to Varicella Zoster Virus (VZV), she should be given Varicella Zoster Immune Globulin (VariZIG) within 10 days of exposure.  However, it’s important to first assess your immunity to VZV. If you’re unsure about your immunity status, you should get tested as soon as possible.  Please consult with your healthcare provider for personalized advice. This information is based on general guidelines and may not be applicable to all situations. It’s always best to seek professional medical advice in such circumstances. Stay safe! | |
|  | Accuracy: 4 | Completeness: 6 | Accuracy:5 | Completeness: 6 | Accuracy: 5 | Completeness: 5 | Accuracy:4 | Completeness: 3 | Accuracy: 5 | Completeness: 6 | Accuracy:5 | Completeness: 6 | Accuracy: 5 | Completeness: 6 | Accuracy: 4 | Completeness: 4 |
| My patient tested positive for Bordetella pertussis (whooping cough). Do I need prophylaxis? | If a patient tests positive for Bordetella pertussis, prophylaxis is recommended for healthcare personnel who have been in contact with the patient, particularly if they are in contact with individuals at risk for severe disease, such as hospitalized neonates, newborn infants, and patients with chronic respiratory conditions. This recommendation comes from the Advisory Committee on Immunization Practices (ACIP).[1] The recommended antimicrobial agents for postexposure prophylaxis include azithromycin, clarithromycin, erythromycin, and trimethoprim-sulfamethoxazole (TMP-SMX).[1] For the patient, the Chinese Thoracic Society (CTS) Asthma Consortium recommends early treatment with macrolides once pertussis is diagnosed, as this can reduce the severity of the disease.[2] However, antibiotics are not recommended for patients with pertussis in the non-catarrhal phase (protracted phase).[2] The American College of Chest Physicians (ACCP) also recommends that patients with confirmed or probable whooping cough should receive a macrolide antibiotic and should be isolated for 5 days from the start of treatment.[3] | | According to the guidelines from the Chinese Thoracic Society (CTS) Asthma Consortium and the American College of Chest Physicians (ACCP), once a diagnosis of pertussis is confirmed, early treatment with macrolides should be initiated.[1-2] The ACCP recommends that patients with confirmed or probable whooping cough should receive a macrolide antibiotic and should be isolated for 5 days from the start of treatment.[2] This is because early treatment within the first few weeks can diminish the coughing paroxysms and prevent spread of the disease.[2] However, the CTS Asthma Consortium notes that antibiotics are not recommended for patients with pertussis in the non-catarrhal phase (protracted phase).[1] Corticosteroids, β2-adrenergic receptor agonists, pertussis specific immunoglobulins, and antihistamines are also not recommended.[1] While these guidelines provide a general approach to the management of pertussis, the specific recommendations from the United States Centers for Disease Control and Prevention (CDC) should be consulted for the most appropriate prophylaxis in the U.S. context. | | I am not a medical professional, but I can offer some general information. It's important to consult with a healthcare professional for advice tailored to your specific situation.  Prophylaxis for whooping cough (caused by Bordetella pertussis) is typically recommended for close contacts of individuals diagnosed with the infection. The goal is to prevent the spread of the bacteria to vulnerable individuals, such as infants, pregnant women, and individuals with weakened immune systems.  Post-exposure prophylaxis with antibiotics like azithromycin or clarithromycin is commonly recommended for close contacts, especially within the first three weeks of exposure. However, the specific recommendations may vary based on factors such as the age and health status of the contacts.  If you've been in close contact with a patient who has tested positive for whooping cough, it is crucial to consult with a healthcare professional promptly. They can provide guidance on whether prophylaxis is necessary for you and discuss any specific considerations based on your health and circumstances. | | I'm not a doctor, but I can provide some general information on this topic.  Prophylaxis for bordetella pertussis, or whooping cough, is typically recommended for close contacts of a person who has tested positive for the disease. This is especially important for individuals who are at a higher risk of severe complications from whooping cough, such as infants, pregnant women, and individuals with weakened immune systems.  The specific recommendations for prophylaxis may vary depending on factors like age and vaccination status. It's important to consult with a healthcare professional, such as the United States Centers for Disease Control and Prevention (CDC) or your local health department, to get specific guidance on whether prophylaxis is necessary for you as a healthcare provider and to determine the appropriate course of action.  Remember that following CDC recommendations and consulting with healthcare authorities is crucial to ensure the best course of action in situations like this. | | As a language model, I can provide general information, but it's important to consult with a healthcare professional for personalized medical advice. Bordetella pertussis, the bacterium that causes whooping cough, is highly contagious. Prophylaxis, typically with antibiotics, is often recommended for close contacts of a person infected with Bordetella pertussis, especially in certain high-risk groups. These groups include:  1. **Infants and Young Children:** They are at higher risk for severe complications from whooping cough. 2. **Pregnant Women:** Especially in the third trimester, as they could pass the infection to their newborn. 3. **Healthcare Workers:** Due to their close contact with vulnerable patients. 4. **Individuals with Pre-existing Health Conditions:** Such as those with compromised immune systems or pre-existing respiratory conditions.  The Centers for Disease Control and Prevention (CDC) often recommends antibiotics for close contacts of a person with whooping cough, irrespective of their vaccination status, to prevent the spread of the disease.  However, the decision for prophylaxis should be based on various factors such as your vaccination status, your health condition, and the nature of your exposure to the patient. Please consult with a healthcare provider or public health official for guidance tailored to your specific situation. | | According to the CDC guidelines, postexposure prophylaxis (PEP) for Bordetella pertussis (whooping cough) is primarily recommended for certain high-risk groups and in specific settings. Here are the key points to consider: Household Contacts: All household contacts of a patient with pertussis should receive PEP, regardless of their immunization status, if they are asymptomatic and within 21 days of the onset of cough in the index patient. This is due to the high secondary attack rates within families. High-Risk Individuals: PEP should be provided to individuals who are at high risk of developing severe illness from pertussis or those who will have close contact with high-risk individuals. This includes: Infants and women in their third trimester of pregnancy. People with pre-existing health conditions that may be exacerbated by a pertussis infection, such as immunocompromised individuals or those with moderate to severe medically treated asthma. Those in close contact with individuals at high risk, such as infants under 12 months of age or pregnant women. Individuals in high-risk settings like neonatal intensive care units, childcare settings, and maternity wards. Closed Settings with Small Case Counts: In closed settings with a small number of identified cases and no ongoing community-wide outbreak, broader use of PEP may be appropriate. Antimicrobial Agents: The preferred agents for both treatment and PEP are macrolides, specifically azithromycin, clarithromycin, and erythromycin. For infants under one month, azithromycin is preferred due to safety concerns with erythromycin. Contraindications and Side Effects: It's important to consider any contraindications to macrolide use and potential side effects, such as gastrointestinal issues and rashes, especially with erythromycin. As a healthcare professional, you should evaluate these considerations in the context of your patient's specific situation, including the infectiousness of the patient with pertussis, the degree of your exposure, and your own health status or risk factors for severe pertussis.  For more detailed information, please refer to the CDC's guidelines on Pertussis and Postexposure Antimicrobial Prophylaxis (PEP)​​ and their recommendations on antimicrobial agents for treatment and PEP of pertussis​​​​. | | According to the CDC, antibiotic prophylaxis is recommended for household contacts of someone with pertussis and for those exposed to pertussis who are at high risk of severe illness or in close contact with someone at high risk. High risk people include infants, women in their third trimester of pregnancy, people with pre-existing health conditions that may be exacerbated by a pertussis infection, and people who have close contact with people who are at high risk. You should also use standard precautions and place your patient in droplet precautions to prevent transmission of B. pertussis in healthcare settings. If you have any questions or concerns, please consult your local health department or infectious disease specialist. | | According to the United States Centers for Disease Control and Prevention (CDC), postexposure antimicrobial prophylaxis (PEP) is recommended in certain situations after exposure to Bordetella pertussis, the bacterium that causes whooping cough. Here are the key points:  The primary objective of PEP is to prevent death and serious complications from pertussis in people at increased risk of severe disease. CDC supports targeting postexposure antibiotic use to people at high risk of developing severe pertussis, as well as people who will have close contact with others at high risk of developing severe pertussis. PEP should be provided to all household contacts of a pertussis case, as secondary attack rates are high, even when household contacts are current with pertussis immunizations. Administration of antimicrobial prophylaxis to asymptomatic household contacts within 21 days of onset of cough in the index patient can prevent symptomatic infection1. High-risk people within 21 days of exposure to an infectious pertussis case should also receive PEP. High-risk people include infants, women in their third trimester of pregnancy, people with pre-existing health conditions that may be exacerbated by a pertussis infection, and people who have close contact with people who are at high risk. For healthcare personnel, regardless of vaccination status, who have an exposure to pertussis and are likely to interact with persons at increased risk for severe pertussis, postexposure prophylaxis is recommended. Please consult with a healthcare professional for personal medical advice. This information is intended for general knowledge and educational purposes. It’s always best to consult with a healthcare provider for specific medical advice or treatments. | |
|  | Accuracy: 5 | Completeness: 5 | Accuracy:5 | Completeness: 6 | Accuracy: 5 | Completeness: 6 | Accuracy:4 | Completeness: 4 | Accuracy: 4 | Completeness: 4 | Accuracy:5 | Completeness: 6 | Accuracy: 1 | Completeness: 1 | Accuracy:5 | Completeness: 3 |
| Patient Exposure | | | | | | | | | | | | | | | | |
| My patient's roommate tested positive for candida auris. What do I need to do for my patient? | In the case of a patient whose roommate has tested positive for Candida auris, a multidrug-resistant yeast known to spread in healthcare settings, several precautions are recommended. These include the implementation of contact precautions, thorough environmental cleaning and disinfection, infection control assessments, contact tracing, and screening of contacts to assess for colonization.[1] Contact precautions involve the use of personal protective equipment, such as gloves and gowns, when interacting with the patient. Environmental cleaning and disinfection should be thorough and frequent, given that Candida auris can persist in the healthcare environment and colonize skin.[1] Infection control assessments should be conducted to identify potential sources of transmission and areas for intervention. Contact tracing and screening of contacts, including the patient in question, are crucial to assess for colonization and limit the spread of Candida auris.[1] It is also recommended to notify public health agencies promptly and conduct a retrospective review of microbiology records and prospective surveillance for cases at laboratories to further limit the spread of C. auris.[1] These measures, as outlined in the Clinical Infectious Diseases publication, are necessary to manage cases and control transmission of C. auris in healthcare facilities.[1] | | In the event of a patient's roommate testing positive for Candida auris, the United States Centers for Disease Control and Prevention (CDC) recommends a series of actions to prevent the spread of this multidrug-resistant yeast. Firstly, the healthcare provider should notify the relevant public health agencies. Secondly, the implementation of contact precautions is crucial. This includes the use of personal protective equipment, such as gowns and gloves, when caring for the patient. Environmental cleaning and disinfection should be thorough, as C. auris can persist in the healthcare environment. This includes cleaning of all surfaces and medical equipment with a disinfectant effective against Clostridium difficile spores. Infection control assessments should be conducted to identify any potential breaches in infection prevention and control. Contact tracing and screening of contacts to assess for colonization is also recommended. This involves testing of close contacts, including roommates, to identify any additional cases. Lastly, a retrospective review of microbiology records and prospective surveillance for cases at laboratories should be conducted to identify any previously unrecognized cases and to monitor for new cases. These measures are all part of an aggressive intervention strategy to limit the spread of C. auris in healthcare facilities.[1] | | I am not a healthcare professional, but I can offer some general information that might be helpful. It's crucial to consult with a qualified healthcare professional for advice tailored to your specific situation. Candida auris is a type of yeast that can cause serious infections, especially in healthcare settings. If your patient's roommate has tested positive for Candida auris, it's important to take appropriate precautions to prevent the spread of the infection. Here are some general recommendations: 1. **Isolation and Precautions:** - Follow strict isolation protocols as recommended by your healthcare facility. This may involve placing the patient in a single room or using contact precautions.  - Use personal protective equipment (PPE) such as gloves and gowns when caring for the patient. 2. **Hand Hygiene:**- Emphasize the importance of thorough hand hygiene for both healthcare providers and the patient. Use soap and water or an alcohol-based hand sanitizer. 3. **Environmental Cleaning:** - Ensure that the patient's environment is regularly cleaned and disinfected, paying special attention to high-touch surfaces. 4. **Screening and Testing:** - Consider screening the patient for Candida auris, especially if they are at a higher risk of infection. Testing can help in early detection and management. 5. **Antifungal Treatment:**- If the patient is diagnosed with Candida auris, the healthcare provider will determine the appropriate antifungal treatment based on the specific circumstances. 6. **Communication:**- Keep open lines of communication with the healthcare team, including infection control specialists, to ensure a coordinated response. 7. **Patient Education:** - Educate the patient about the importance of adhering to infection control measures and any prescribed treatments. It's crucial to follow the guidelines and recommendations provided by your healthcare facility and local health authorities. Consult with the infectious disease team or infection control specialists at your hospital for specific guidance based on your patient's condition and the facility's protocols. | | I'm not a medical professional, but I can offer some general information based on the CDC's recommendations for Candida auris. It's important to consult with a healthcare provider for specific guidance regarding your patient's situation. However, here are some general steps to consider:  Isolate the Patient: If your patient is in a healthcare facility, they should be placed in a single room with a private bathroom. Healthcare workers should follow strict infection control precautions when caring for the patient. Screening and Testing: Your patient may need screening and testing for Candida auris to determine if they are colonized or infected. Swab samples from different body sites may be taken for testing. Notify Healthcare Facilities: If your patient has been in other healthcare facilities recently, notify those facilities about the Candida auris case. Enhanced Cleaning and Disinfection: Healthcare facilities should implement enhanced cleaning and disinfection protocols to prevent the spread of Candida auris. Education: Educate your patient and their caregivers about proper hygiene practices and infection prevention measures. Follow CDC Guidelines: Continue to follow the latest guidelines and recommendations from the CDC regarding Candida auris. Please consult with a healthcare professional or the CDC for specific guidance tailored to your patient's situation. | | Candida auris is a significant healthcare concern due to its resistance to multiple antifungal drugs and its ability to cause outbreaks in healthcare settings. Here are the general steps to take if your patient's roommate has tested positive for Candida auris: 1. **Infection Control Measures**: Implement strict infection control measures immediately. This includes using personal protective equipment (PPE) like gloves and gowns when entering the patient's room. 2. **Patient Monitoring**: Monitor your patient closely for any signs of infection, especially if they have wounds, catheters, or other medical devices. Symptoms of Candida auris infection can be similar to other infections and might include fever and chills that don't improve with antibiotics. 3. **Environmental Cleaning and Disinfection**: Ensure thorough and frequent cleaning and disinfection of the patient's room and nearby areas. Candida auris can survive on surfaces for long periods, so environmental hygiene is critical. 4. **Testing and Surveillance**: Consider testing your patient for Candida auris, especially if they are at high risk (e.g., immunocompromised, recent surgery, long-term antibiotic use). Coordination with infection control and microbiology departments is essential for surveillance and management. 5. **Cohorting Patients**: If possible, cohort patients with Candida auris separately from those without the infection to prevent cross-contamination. 6. **Education and Communication**: Educate healthcare staff and the patient's visitors about Candida auris, its transmission, and the importance of hand hygiene and other preventive measures. 7. **Reporting and Coordination with Health Authorities**: Report the case to the appropriate health authorities and follow any guidelines or recommendations they provide. 8. **Review and Adjust Treatment**: If your patient does develop an infection, you'll need to work closely with infectious disease specialists to choose the most effective antifungal treatment, considering Candida auris's drug-resistant nature.  Remember, each healthcare setting may have specific protocols and guidelines for managing exposure to multidrug-resistant organisms like Candida auris. Always follow your facility's policies and consult with infection control experts for tailored advice. | | In managing a situation where a patient's roommate has tested positive for Candida auris, it's important to follow the guidelines provided by the United States Centers for Disease Control and Prevention (CDC). Here are the general steps you should consider:  Infection Control Measures: Implement standard and contact precautions. Use personal protective equipment (PPE) when caring for the patient, including gloves and gowns. This is to prevent the spread of Candida auris. Patient Testing: Consider testing your patient for Candida auris, especially if they have signs or symptoms of infection, or if they have been in close contact with the infected roommate. Environmental Cleaning and Disinfection: Ensure thorough cleaning and disinfection of the patient's environment. Candida auris can persist on surfaces, so it's important to use EPA-registered disinfectants that are effective against fungal pathogens. Monitoring and Reporting: Monitor the patient for any signs of infection. If Candida auris is suspected or confirmed, report it to the appropriate healthcare authorities as required. Education and Awareness: Educate healthcare staff and the patient (if appropriate) about Candida auris, its transmission, and prevention strategies. Cohorting Patients: If possible, cohort patients with Candida auris together and assign dedicated staff to care for these patients to reduce the risk of transmission. Antifungal Stewardship: If the patient develops an infection and requires antifungal treatment, consult with an infectious disease specialist. Candida auris can be resistant to multiple antifungal drugs, so appropriate antifungal stewardship is crucial.  Remember, these are general guidelines. It's crucial to consult with infection control experts at your facility and adhere to the specific protocols and recommendations of your healthcare institution and the CDC. Regularly check the CDC's website for the most up-to-date guidelines on handling Candida auris infections. | | Candida auris is a type of fungus that can cause serious illness, especially in hospitalized patients or those with weakened immune systems. Here are some precautions you can take for your patient who has been exposed to Candida auris:  Hand Hygiene: Healthcare personnel should follow standard hand hygiene practices. Alcohol-based hand sanitizer is the preferred method for C. auris when hands are not visibly soiled. If hands are visibly soiled, wash with soap and water.  Transmission-Based Precautions & Room Placement: Use Contact Precautions to manage patients with C. auris in acute care hospitals and long-term acute care hospitals.  Cleaning and Disinfecting: The patient care environment and reusable equipment should be cleaned and disinfected with recommended products, including shared mobile equipment.  Communication: Communicate about the patient’s C. auris status when the patient is transferred.  Screening: Screen contacts of newly identified case patients to identify C. auris colonization.  Laboratory Surveillance: Conduct laboratory surveillance of clinical specimens to detect additional cases.  Remember, wearing gloves is not a substitute for hand hygiene. Also, decisions to discharge the patient from one level of care to another should be based on clinical criteria and the ability of the accepting facility to provide care, not on the presence or absence of infection or colonization.  Please consult with your healthcare facility’s infection control team for more specific guidance. It’s also important to stay updated with the latest guidelines from the CDC or other relevant health organizations. | | According to the United States Centers for Disease Control and Prevention (CDC), here are the key steps you should take for your patient who has been exposed to Candida auris:  Adherence to hand hygiene: Healthcare personnel should follow standard hand hygiene practices. Alcohol-based hand sanitizer is the preferred method for C. auris when hands are not visibly soiled. If hands are visibly soiled, wash with soap and water.  Use of Transmission-Based Precautions: Healthcare providers should use Contact Precautions to manage patients with C. auris in acute care hospitals and long-term acute care hospitals.  Cleaning and disinfecting the patient care environment: This includes daily and terminal cleaning and disinfecting of the patient’s room and reusable equipment with recommended products.  Communication about patient’s C. auris status: Ensure to communicate about the patient’s C. auris status when the patient is transferred.  Screening contacts of newly identified case patients: This is to identify C. auris colonization.  Laboratory surveillance of clinical specimens: This is to detect additional cases.  Remember, these are general guidelines and specific situations may require additional measures. Always consult with a healthcare professional or infection control specialist for advice tailored to your specific circumstances. Please refer to the CDC’s guidelines for more detailed information. Stay safe! | |
|  | Accuracy: 5 | Completeness: 6 | Accuracy:5 | Completeness: 6 | Accuracy: 5 | Completeness: 6 | Accuracy: 5 | Completeness: 6 | Accuracy: 5 | Completeness: 6 | Accuracy:5 | Completeness: 6 | Accuracy: 5 | Completeness: 6 | Accuracy: 5 | Completeness: 6 |
| My patient had a roommate that tested positive for COVID-19. What do I need to do for my patient? | For a patient who had a roommate test positive for COVID-19, several precautions should be taken to prevent potential transmission. The patient should be tested for SARS-CoV-2, given the high transmission rate in shared rooms.[1] The European Society for Medical Oncology recommends that patients wear personal protective equipment (PPE) during treatment, and healthcare workers should also use PPE meticulously, including eye protection, a gown, a surgical mask or a filtering face piece (FFP), and gloves.[2] The International Cardio-Oncology Society suggests that healthcare systems adopt a universal face mask policy, with an increased level of respiratory protection for healthcare personnel in high-risk exposure scenarios. Hand hygiene is also crucial, with hand washing before and after seeing and examining a patient.[3] The Surviving Sepsis Campaign recommends the use of negative pressure rooms for aerosol-generating procedures, and if this is not feasible, a portable HEPA filter should be used in the room wherever possible.[4] In addition, the patient should be monitored for symptoms of COVID-19, and if symptoms develop, they should be isolated and treated accordingly. Regular cleaning of examination rooms and instruments for clinical examination is also recommended to minimize infection risks.[3] | | If a patient's roommate has tested positive for COVID-19, the United States Centers for Disease Control and Prevention (CDC) recommends that the patient should be considered a close contact and should quarantine. The patient should stay away from others, especially those who are at higher risk for getting very sick from COVID-19, for 14 days after their last contact with their roommate. The patient should monitor their health and watch for symptoms of COVID-19, such as fever, cough, shortness of breath, or other symptoms. If symptoms develop, they should seek medical attention and get tested for COVID-19. The patient should also follow good hygiene practices, such as washing hands often and avoiding touching their face. They should wear a mask when around others, and clean and disinfect frequently touched surfaces daily. It's important to note that these recommendations may change as new information about COVID-19 becomes available, so it's crucial to stay updated with the latest guidelines from the CDC. | | I am not a healthcare professional, but I can offer some general guidance. It's crucial to consult with a healthcare professional or follow your organization's protocols for specific advice tailored to your patient's situation. However, here are some general steps that may be recommended: 1. **Isolate the Patient:** If possible, move the patient to a separate room to reduce the risk of transmission. If a separate room is not available, try to maintain a safe distance from the roommate. 2. **Notify Healthcare Providers:** Inform the relevant healthcare providers about the situation, including the patient's primary care physician or the healthcare facility where they are receiving care. 3. **Testing:** Arrange for COVID-19 testing for the patient, especially if they start exhibiting symptoms or if it's recommended by healthcare authorities. Follow the guidelines provided by local health authorities for testing procedures. 4. **Monitor Symptoms:** Keep a close eye on the patient for any symptoms of COVID-19, such as fever, cough, shortness of breath, or other respiratory symptoms. Report any changes to healthcare professionals promptly. 5. **Quarantine and Follow Guidelines:** Follow the guidelines and recommendations provided by local health authorities and healthcare professionals regarding quarantine and isolation protocols. This may involve staying at home, avoiding contact with others, and practicing good hygiene. 6. **Personal Protective Equipment (PPE):** Ensure that both the patient and any caregivers or healthcare workers interacting with the patient use appropriate PPE, such as masks, gloves, and gowns, to minimize the risk of transmission. 7. **Clean and Disinfect:** Regularly clean and disinfect surfaces in the patient's environment, especially commonly-touched surfaces like doorknobs, light switches, and countertops. 8. **Communicate with Roommate:** If possible, communicate with the roommate and their healthcare providers to share information about the situation and take necessary precautions to prevent further spread. Remember, it's crucial to consult with healthcare professionals or follow your organization's protocols for the most accurate and specific guidance tailored to your patient's circumstances. | | I'm not a medical professional, but I can try to provide some general information based on the recommendations of the United States Centers for Disease Control and Prevention (CDC). If your patient had a roommate who tested positive for COVID-19, here are some general steps your patient may consider: Isolation and Quarantine: If your patient had close contact with a COVID-19 positive roommate, they should consider isolating themselves to prevent potential spread. Isolation means staying away from others even within the same household. Quarantine may be necessary for 14 days to monitor for symptoms. Monitoring for Symptoms: Your patient should monitor themselves for COVID-19 symptoms, which may include fever, cough, shortness of breath, loss of taste or smell, and other flu-like symptoms. If any symptoms develop, they should contact their healthcare provider and get tested for COVID-19. Testing: It's advisable for your patient to get tested for COVID-19, especially if they develop symptoms or if they were in close contact with the COVID-19 positive roommate. Follow CDC Guidelines: Your patient should follow any guidance provided by the CDC regarding quarantine, isolation, and testing. These guidelines may change over time, so it's essential to stay updated on the latest recommendations. Inform Healthcare Provider: If your patient has a healthcare provider, they should inform them about the potential exposure to COVID-19 so that their healthcare provider can provide appropriate guidance and care. Practice Preventive Measures: Your patient should continue to practice preventive measures such as wearing a mask, maintaining physical distance, frequent handwashing, and avoiding close contact with others, even within the household. It's crucial for your patient to consult with their healthcare provider for personalized advice based on their specific situation. Additionally, please refer to the most recent CDC guidelines and recommendations for the most up-to-date information on COVID-19 management. | | If your patient had a roommate who tested positive for COVID-19, there are several steps you should take to ensure their safety and the safety of others: 1. **Isolate the Patient:** Move your patient to a separate room, if possible, to prevent further exposure. Isolation helps limit the spread of the virus. 2. **Testing for COVID-19:** Arrange for your patient to be tested for COVID-19, even if they are not showing symptoms. Follow the guidelines of your local health authority regarding testing protocols. 3. **Monitor for Symptoms:** Keep a close watch for any symptoms of COVID-19 in your patient, such as fever, cough, difficulty breathing, loss of taste or smell, etc. Regularly check their temperature and respiratory status. 4. **Use Personal Protective Equipment (PPE):** Ensure that all healthcare workers and caregivers interacting with the patient use appropriate PPE, including masks, gloves, and gowns, to prevent transmission. 5. **Follow Infection Control Protocols:** Adhere strictly to infection control protocols in your healthcare setting. This includes regular hand washing, using hand sanitizers, and disinfecting surfaces and equipment frequently. 6. **Educate the Patient:** Inform your patient about the situation and the importance of following isolation guidelines, wearing a mask, and practicing good hygiene. 7. **Contact Tracing:** Notify public health authorities so they can begin contact tracing and notify others who may have been exposed. 8. **Adhere to Public Health Guidelines:** Follow the advice and guidelines of your local public health authority for managing COVID-19 exposures in healthcare settings. 9. **Mental Health Support:** Remember that isolation and the fear of COVID-19 can be stressful. Provide support to your patient and consider referring them for mental health support if needed. 10. **Re-Evaluate Regularly:** Continue to monitor the situation and be prepared to adjust your approach based on the patient's health status and evolving public health guidelines.  It's crucial to stay updated with the latest guidelines from health authorities, as recommendations may change based on new information about the virus. | | When managing a patient who has been exposed to someone who tested positive for COVID-19, it's important to follow the guidelines established by the United States Centers for Disease Control and Prevention (CDC). As of my last update in April 2023, here are some general steps you should consider, but please verify with the latest CDC guidelines as recommendations may have changed: Quarantine and Isolation: If your patient is not fully vaccinated or hasn't received a booster dose, they should quarantine. This typically means staying at home and avoiding contact with other people for a specified period, often around 5 days. The duration and specifics can vary based on the latest CDC guidelines and the vaccination status of the patient. Testing for COVID-19: It's recommended that the exposed individual gets tested for COVID-19. The timing of the test may vary, but it's generally advised to test a few days after exposure or immediately if symptoms develop. Monitoring Symptoms: Your patient should monitor for COVID-19 symptoms, which can include fever, cough, shortness of breath, and others. If they develop symptoms, they should get tested and follow isolation guidelines. Preventive Measures: Regardless of testing and quarantine, it's important to continue practicing preventive measures such as wearing masks, maintaining social distancing, and frequent handwashing. Consultation and Further Steps: If your patient is at higher risk for severe illness from COVID-19 due to age or underlying health conditions, it's important to consult with healthcare professionals for personalized advice and possibly more stringent precautions. Vaccination Status: The recommendations might differ depending on whether your patient is fully vaccinated and boosted. Fully vaccinated individuals might have different guidelines regarding quarantine and testing. Informing Close Contacts: It's also advised to inform any close contacts of your patient about the potential exposure, so they can also take necessary precautions. Follow Local Health Guidelines: Additionally, adhere to any specific recommendations or mandates from local health authorities, as they might have more specific guidelines tailored to the local situation.  Remember, these guidelines can change as new information about the virus emerges, so it's crucial to stay updated with the latest information from the CDC and other reliable health sources. | | If your patient’s roommate has tested positive for COVID-19, here are some steps you can take:  1. Isolation: The patient should isolate from others, regardless of their vaccination status. If they are sick and suspect that they have COVID-19 but do not yet have test results, they should also isolate. If the patient tests positive, they should follow the full isolation recommendations.  2. Testing: The patient should get tested at least five days after being exposed, or sooner if they have symptoms.  3. Masking: The patient should wear a high-quality mask for 10 days while indoors in public areas. 4. Monitor Symptoms: The patient should monitor their symptoms. If they have an emergency warning sign (like trouble breathing), they should seek emergency medical care immediately. 5. Treatment: If the patient tests positive for COVID-19, there may be additional treatments available to them. Remember, these recommendations are meant to supplement—not replace—any federal, state, local, territorial, or tribal health and safety laws, rules, and regulations. Always consult with healthcare professionals for the most accurate information. Stay safe! | | Centers for Disease Control and Prevention Explore According to the United States Centers for Disease Control and Prevention (CDC), if your patient was exposed to someone who tested positive for COVID-19, here are the steps you should take:  Start Precautions Immediately: Your patient should wear a mask as soon as they find out they were exposed. Continue Precautions for 10 Full Days: Your patient can still develop COVID-19 up to 10 days after they have been exposed1. They should wear a high-quality mask or respirator (e.g., N95) any time they are around others inside their home or indoors in public. Get Tested on Day 6: Your patient should get tested at least 5 full days after their last exposure. They should test even if they don’t develop symptoms. If the Test is Negative: Your patient should continue taking precautions through day 10. If the Test is Positive: Your patient should isolate immediately. Please note that these guidelines are subject to change as new information about the virus becomes available. Always refer to the latest guidelines from the CDC. If your patient develops severe symptoms, seek medical attention immediately. Stay safe! | |
|  | Accuracy: 3 | Completeness: 3 | Accuracy: 4 | Completeness: 5 | Accuracy: 4 | Completeness: 6 | Accuracy:5 | Completeness: 5 | Accuracy: 5 | Completeness: 6 | Accuracy: 4 | Completeness: 4 | Accuracy: 4 | Completeness: 4 | Accuracy:5 | Completeness: 4 |
| Environmental Cleaning | | | | | | | | | | | | | | | | |
| My patient came to clinic and was found to have bed bugs. What do we need to do with the clinic room? | If a patient is found to have bed bugs in a clinic room, the room should be thoroughly cleaned and treated to eliminate the infestation. This typically involves a combination of non-chemical and chemical methods. Non-chemical methods include vacuuming, laundering of bedding and clothing at high temperatures, and the use of heat or cold treatments. Chemical methods involve the use of approved insecticides, which should be applied by a professional pest control service to ensure safety and effectiveness. In addition to treating the room, it's important to consider the patient's home environment, as this is likely the source of the infestation. The patient should be educated about bed bugs, including how to identify them and how to prevent further infestations. They should be advised to seek professional pest control services for their home. While the reference provided discusses the contamination of hospital rooms with healthcare-associated pathogens, it does not specifically address the issue of bed bugs. However, the principles of thorough cleaning and disinfection, as well as the importance of considering the source of contamination, are broadly applicable to the management of bed bugs in a clinic setting. No directly relevant references were found to provide a more specific guideline on this issue. However, the general principles of pest control and infection prevention should guide the response to a bed bug infestation in a clinic room. | | No results were found in the high quality medical literature. This could be because no such peer-reviewed medical evidence exists, or because it does not meet the necessary quality threshold to be cited by OpenEvidence. If you believe this is an error, please help us improve OpenEvidence and let us know at help@openevidence.com. | | Dealing with a bed bug infestation in a clinic room requires prompt and thorough action to ensure the safety of patients and staff. Here are steps you can take to address the issue: 1. **Isolate the Infested Area:**- Close off the affected room to prevent the bugs from spreading to other areas.  - Inform clinic staff about the situation and restrict access to the infested room until the issue is resolved. 2. **Notify Management and Staff:**- Inform clinic management and staff about the bed bug infestation.  - Provide information on the steps that will be taken to address the problem and any temporary changes in clinic operations. 3. **Contact Professional Pest Control:** - Engage a licensed pest control professional experienced in treating bed bug infestations.  - Ensure that the chosen pest control method is safe for healthcare environments and complies with any relevant regulations. 4. **Inspect and Treat Furniture and Bedding:**- Inspect all furniture, including chairs, couches, and beds, in the affected room.  - Remove and treat infested furniture and bedding appropriately, following professional advice. 5. **Thorough Cleaning:** - Vacuum the entire room, paying special attention to seams, crevices, and cracks where bed bugs may hide.  - Dispose of the vacuum bag in a sealed plastic bag outside the clinic.  - Launder all linens, curtains, and other washable items in hot water and dry them on the highest heat setting. 6. **Seal and Dispose:**- Seal any cracks or gaps in walls, floors, and furniture where bed bugs might hide.  - Discard items that cannot be treated or cleaned, if necessary. 7. **Monitor and Prevent Reinfestation:** - Regularly monitor the clinic room for signs of bed bugs, even after treatment.  - Implement preventive measures, such as regularly inspecting furniture and educating staff on how to identify and report bed bug sightings. 8. **Educate Staff and Patients:**- Provide information to clinic staff about bed bug prevention, early detection, and reporting procedures.  - Educate patients on recognizing bed bug bites and encourage them to report any concerns. 9. **Document the Incident:**- Keep detailed records of the bed bug incident, including dates of discovery, actions taken, and communication with pest control professionals. 10. **Follow Up:** - Schedule follow-up inspections with the pest control professional to ensure that the infestation has been completely eradicated. Remember, addressing a bed bug infestation in a healthcare setting requires a coordinated effort between clinic staff and professional pest control services to minimize any potential health risks. Always prioritize the safety and well-being of patients and staff during the process. | | I'm not a medical professional, but I can provide some general guidance on how to handle a situation where a patient with bed bugs has been in a clinic room. It's important to follow proper protocols to prevent the spread of bed bugs. Here are some steps you can consider: Isolate the affected area: Isolate the clinic room where the patient was found to have bed bugs. Ensure that no one else enters the room until it has been properly treated. Inform staff and patients: Inform clinic staff and patients who may have been in contact with the affected room about the situation. Encourage them to monitor their belongings for any signs of bed bugs. Contact a pest control professional: It's crucial to contact a licensed pest control professional who specializes in bed bug extermination. They can assess the extent of the infestation and develop a treatment plan. Treatment of the clinic room: The pest control professional will recommend the appropriate treatment for the clinic room. This may involve using pesticides, heat treatments, or other methods to eliminate bed bugs. Launder and inspect linens and clothing: Any linens, clothing, or fabrics in the clinic room should be laundered at high temperatures to kill any bed bugs and their eggs. Inspect and ensure that they are free of bed bugs before reuse. Vacuum and clean: Vacuum the affected room thoroughly, paying attention to cracks and crevices where bed bugs may hide. Dispose of the vacuum bag or clean the vacuum thoroughly afterward. Monitor and prevent reinfestation: After treatment, continue to monitor the clinic room and surrounding areas for any signs of bed bugs. Implement preventive measures to reduce the risk of future infestations, such as regular inspections and education for staff on how to recognize bed bugs. It's essential to consult with a pest control professional and possibly involve healthcare facility management to ensure that the proper procedures are followed in your specific clinic setting. Additionally, following any guidelines or recommendations from the United States Centers for Disease Control and Prevention (CDC) is crucial to ensure the safety and well-being of staff and patients. Please consult with a healthcare professional or pest control expert for specific advice tailored to your clinic's situation. | | Dealing with bed bugs in a clinical setting requires a multi-step approach to ensure the infestation is fully addressed and to prevent its spread. Here are the steps you should consider:  1. **Isolate the Room**: Immediately close off the room where the bed bugs were found to prevent them from spreading to other areas. 2. **Notify Facility Maintenance**: Contact your facility's maintenance or housekeeping department. They will likely have protocols in place for such situations. 3. **Professional Pest Control**: It is crucial to get a professional pest control service involved. They can assess the extent of the infestation and treat the area effectively. Over-the-counter insecticides are usually not effective against bed bugs. 4. **Thorough Cleaning**: After the pest control treatment, the room should be thoroughly cleaned. This includes washing any fabric items in hot water and vacuuming the area. The vacuum bag should be disposed of immediately in a sealed plastic bag. 5. **Inspect Adjacent Areas**: It's important to inspect adjacent rooms and areas to ensure that the infestation has not spread. 6. **Follow-Up Treatments**: Bed bugs can be quite resilient, so follow-up treatments by the pest control professionals may be necessary. 7. **Educate Staff**: Inform your staff about the situation and educate them on how to identify bed bugs and the proper protocols if they suspect an infestation in the future. 8. **Monitor for Recurrence**: After the initial treatment, keep monitoring the area for any signs of bed bugs returning. 9. **Document the Incident**: Keep a record of the infestation and the steps taken to resolve it. This documentation can be useful for future reference and for regulatory compliance. 10. **Patient Care and Communication**: Consider the implications for the patient who was found to have bed bugs. They may need guidance on how to manage the infestation in their home and reassurance about the stigma often associated with bed bugs.  Remember, the key is to act quickly and thoroughly to prevent the spread of bed bugs and to maintain a safe and clean environment for patients and staff. | | When dealing with bed bugs in a clinic setting, it's essential to follow the recommendations of public health authorities like the United States Centers for Disease Control and Prevention (CDC). Here are the general steps that are typically recommended: Isolate the Area: Close off the room or area where bed bugs have been found to prevent their spread to other parts of the facility. Notify Facility Maintenance: Immediately inform your facility's maintenance or housekeeping staff. They may have specific protocols for dealing with bed bugs. Professional Pest Control: It is usually recommended to contact a professional pest control company experienced in dealing with bed bugs. They can assess the situation and treat the room effectively. Clean and Vacuum the Room: Thoroughly clean the room, including vacuuming any carpets, furniture, and crevices where bed bugs might hide. However, remember that vacuuming alone won't eliminate bed bugs but can help reduce their numbers. Launder Any Fabric Materials: Wash and dry any fabric materials (like curtains, bedding, or uniforms) at high temperatures. Bed bugs and their eggs are killed by heat. Follow-Up Treatments: Multiple treatments might be necessary. Bed bugs are notoriously hard to eliminate, and repeated treatments may be needed. Educate Staff and Patients: Provide information about bed bugs, their signs, and how to prevent their spread. This can help in early detection and management in the future. Monitoring: After treatment, continue to monitor the area for signs of bed bugs to ensure that the infestation has been fully resolved. Document the Incident: Keep a record of the infestation and the steps taken to resolve it. This can be helpful for future reference and for regulatory compliance. Remember, these are general guidelines. For specific instructions and protocols, it's important to consult with local health authorities and follow the policies of your healthcare facility. Additionally, since bed bug infestations can have implications for public health and hygiene, it's important to handle them promptly and effectively. | | If a patient is found to have bed bugs in a clinic room, here are the recommended steps to follow: Capture a Bed Bug: If possible, capture a bed bug and place it in a sealed container to assist pest management in their assessment. Leave the Room Intact: Do not clean or remove items (e.g., linens, furniture, equipment) from the room. This facilitates the determination of the extent of the infestation and prevents the spread of bugs to other areas. Close Off the Room: Close off the room or area from use and place signage indicating "Do Not Enter, Do Not Remove Equipment, Linen or Furniture from Room". Notify Relevant Personnel: Notify the Housekeeping Supervisor and Nursing Supervisor immediately, and Infection Prevention during normal business hours. Develop a Treatment Plan: It’s best to develop a bed bug prevention and treatment plan for your facility. More than one treatment is typically necessary to fully get rid of a bed bug infestation and prevent them from returning. Clean the Room: After the patient leaves the room or area, terminally clean the room following these procedures: Staff person gowns up and also places disposable booties on feet. Remove all trash and double bag it. Remove linen and double bag. Remove curtain and double bag. Room is to be terminally cleaned with either alcohol, Steri-Fab or steam cleaner. Follow-up Inspections: Once control measures have been implemented, routine follow-up inspections are required to monitor the effectiveness of the treatments.  Remember, bed bugs are hard to find and require special tools and training. It’s best to have professionals set up a treatment plan and provide reliable technology and methods to detect, remove and prevent bed bugs. If a facility experiences periodic introduction of bed bugs, a regular detection program with the help of a pest management professional should be considered. | | According to the Centers for Disease Control and Prevention (CDC) and other resources, here are some steps you can take if a clinic room is found to have bed bugs:  Regular Inspection: The best way to prevent bed bugs is regular inspection for signs of an infestation. Vacuuming: Vacuuming reduces bed bug populations. Clean and vacuum bed bug prone areas daily. Immediately seal and dispose of the vacuum bag. Encasements: Install encasements on mattress and box spring. Staff Training: Facility managers should have a published bed bug protocol, and staff should be trained to recognize bed bugs and evidence of their presence. Intake Procedures: Intake procedures to screen incoming clients and bag their belongings may also help. Isolation of Infested Items: Items suspected of infestation should be contained until they can be removed, sanitized, or disposed of. Follow-up Inspection: Rooms that have been serviced for bed bugs should be rescheduled for follow-up inspection, re-serviced as needed, and kept offline until the bed bugs have been successfully controlled. Remember, bed bugs do not transmit disease, but their presence can cause inconvenience and distress. It’s important to address the issue promptly to prevent further spread. Please consult with a pest control professional for comprehensive treatment. | |
|  | Accuracy: 5 | Completeness: 5 | Accuracy:1 | Completeness: 1 | Accuracy: 5 | Completeness: 6 | Accuracy:5 | Completeness: 6 | Accuracy: 4 | Completeness: 6 | Accuracy:5 | Completeness: 6 | Accuracy: 3 | Completeness: 5 | Accuracy:5 | Completeness: 5 |
| My patient came to clinic and was found to have COVID-19. There was an aerosol-generating procedure performed in the room. What do we need to do with the clinic room? | After performing an aerosol-generating procedure on a patient with COVID-19, several precautions should be taken with the clinic room to prevent cross-contamination. The Surviving Sepsis Campaign recommends the use of negative pressure rooms with a minimum of 12 air changes per hour or at least 160 L/second/patient in facilities with natural ventilation.[1] If this is not feasible, a portable HEPA filter should be used in the room wherever possible.[1] The Surgical Infection Society (SIS) emphasizes the importance of complete cleaning of the room after operating on a patient with COVID-19 infection.[2] Cleaning supplies designated for the COVID-19 OR should be segregated from other supplies used for other ORs. Human coronaviruses can persist on uncleaned surfaces for up to nine days, but can be eliminated with disinfectants containing 70% ethanol, 0.1% sodium hypochlorite, or 0.5% hydrogen peroxide.[2] Disposable equipment or filters must be discarded and never cleaned or disinfected. Non-disposable equipment should be double-bagged in red biohazard bags for transport for centralized cleaning.[2] Personnel involved in the cleaning process must wear full PPE similar to that worn in the OR, and be well trained in the procedures and techniques specific to a COVID-19–positive room.[2] The time for proper decontamination of an OR ranges up to three hours between ending of cleaning and permitting non-protected individuals into an unoccupied room, depending on the filtration rate.[2] The Portuguese Pulmonology Society also recommends that rooms should be unavailable for a period of time allowing decontamination following procedures performed on suspected or confirmed COVID-19 patients; air circulation rooms can be tested in advance to determine this period of time.[3] In summary, the precautions include using negative pressure rooms or HEPA filters, thorough cleaning and disinfection of the room and equipment, and allowing sufficient time for decontamination before the room is used again. | | After an aerosol-generating procedure (AGP) has been performed on a patient with COVID-19, the clinic room requires thorough cleaning and disinfection. The European Society of Paediatric Radiology (ESPR) recommends that the room should be left for at least 20 minutes with the door closed to allow aerosol droplets to settle, and depending on air exchange rates, the room could be out of service for approximately 1 hour after imaging an infected patient.[1] Surfaces, including doorknobs and equipment, should be cleaned with viricidal agents, such as 75% ethanol or chlorine-containing disinfectant.[1] The Surgical Infection Society (SIS) advises that complete cleaning of the room is crucial, using U.S. Environmental Protection Administration-registered hospital detergents or disinfectants. Disposable equipment or filters must be discarded, and non-disposable equipment should be double-bagged for centralized cleaning. Personnel involved in cleaning must wear full personal protective equipment (PPE) and be trained in procedures specific to a COVID-19–positive room.[2] Additionally, the Surviving Sepsis Campaign suggests the use of negative pressure rooms with a minimum of 12 air changes per hour or portable HEPA filters to reduce virus transmission in settings where negative pressure rooms are not available.[3] The Society of Thoracic Surgeons (STS) COVID-19 Task Force recommends a standoff period for aerosol clearance after the patient has left the room, with the duration depending on factors such as room size and ventilation conditions, followed by cleaning with EPA-approved hospital disinfectant.[4] In summary, after an AGP in a COVID-19 patient, the room should be left vacant for a sufficient time to allow aerosol clearance, followed by rigorous cleaning and disinfection with appropriate agents, and adherence to PPE and waste disposal protocols as per guidelines from the ESPR, SIS, Surviving Sepsis Campaign, and STS COVID-19 Task Force.[1-4] | | When a patient with COVID-19 undergoes an aerosol-generating procedure in a clinic room, it is important to take appropriate measures to minimize the risk of transmission to healthcare workers and other patients. Here are general guidelines, but please note that recommendations may vary based on your specific healthcare facility's protocols and local health department guidelines. Always consult with your institution's infection control team for the most accurate and up-to-date information: 1. **Isolation and Cleaning:* - Immediately designate the room as an isolation area.  - Keep the room closed for a sufficient amount of time to allow aerosols to settle.  - Follow your facility's cleaning and disinfection protocols for COVID-19. Use Environmental Protection Agency (EPA)-approved disinfectants with efficacy against SARS-CoV-2. 2. **Personal Protective Equipment (PPE):**- Ensure that all healthcare workers involved in patient care wear appropriate PPE, including N95 respirators or higher-level respirators, eye protection (goggles or face shields), gloves, and gowns. 3. **Air Exchange:** - Increase ventilation in the room by opening windows and doors, if possible.  - Allow time for air exchanges to reduce the concentration of airborne particles. Follow your facility's HVAC guidelines. 4. **Signage:** - Clearly mark the room as a COVID-19 isolation area.  - Provide information on the door regarding the need for appropriate PPE and precautions. 5. **Patient Movement:** - Minimize unnecessary movement of the patient within the facility.  - If the patient needs to be transferred to another location, ensure that proper infection prevention measures are followed during transport. 6. **Healthcare Worker Monitoring:** - Monitor healthcare workers for symptoms of COVID-19 and follow your facility's protocols for testing and quarantine if necessary. 7. **Communication:** - Notify relevant healthcare personnel and departments about the potential exposure, following your facility's reporting procedures. 8. **Review and Update Protocols:** - Regularly review and update infection control protocols based on the latest guidance from health authorities. Always consult with your facility's infection control team or designated personnel to ensure that you are following the specific protocols and guidelines in place at your institution. These measures are crucial to prevent the spread of COVID-19 within healthcare settings. | | I'm not a medical professional, but I can provide some general guidelines based on information available up to my last knowledge update in January 2022. It's crucial to consult with the United States Centers for Disease Control and Prevention (CDC) or a healthcare professional for the most up-to-date and specific guidance regarding COVID-19 protocols. Here are some general steps you might consider: Isolate the Room:Ensure the room is closed off to prevent access by other patients and staff.If possible, keep the room vacant for a specific duration to allow for air exchange and decontamination. Cleaning and Disinfection:Follow CDC guidelines for cleaning and disinfecting healthcare facilities.Use appropriate EPA-registered disinfectants effective against the virus that causes COVID-19. Pay special attention to high-touch surfaces like doorknobs, light switches, and equipment. Ventilation: Ensure proper ventilation in the room by opening windows or using mechanical ventilation systems. Increase the air exchange rate to reduce the concentration of airborne particles. Personal Protective Equipment (PPE): Ensure that healthcare personnel entering the room use appropriate PPE, including masks, gowns, gloves, and eye protection. Monitor and Follow CDC Guidance: Stay updated on the latest CDC guidance for healthcare facilities regarding COVID-19. Consider contacting your local health department for additional guidance or reporting requirements. Quarantine and Testing: Consider quarantining patients and staff who were in the room during or after the aerosol-generating procedure. Offer testing to individuals who may have been exposed. Please consult the CDC's website or contact your local health department for specific recommendations and protocols, as guidelines may have evolved since my last knowledge update. | | If a patient with COVID-19 has undergone an aerosol-generating procedure in a clinic room, certain steps should be taken to ensure safety and prevent the spread of the virus. These include: 1. **Room Ventilation**: The room should be well-ventilated. If the room has windows, they should be opened to allow fresh air to circulate. 2. **Cleaning and Disinfecting**: The room must be thoroughly cleaned and disinfected. This includes all surfaces that the patient may have touched or that could have been exposed to respiratory droplets, such as doorknobs, bedrails, medical equipment, and other high-touch surfaces. 3. **Time Interval Before Reuse**: It's recommended to wait a certain period before allowing others to enter the room. The Centers for Disease Control and Prevention (CDC) provides specific guidelines on the duration, which can vary depending on the room's ventilation rate. 4. **Personal Protective Equipment (PPE)**: Staff involved in cleaning should wear appropriate PPE, including gloves, gowns, and respiratory protection, to prevent self-contamination. 5. **Waste Disposal**: Any disposable items used by the patient or in the cleaning process should be treated as infectious waste and disposed of according to local regulations for biohazardous waste. 6. **Signage and Access Control**: The room should be clearly marked as contaminated, and access should be limited to essential personnel only until the cleaning and disinfection process is complete. 7. **Follow-up with Staff and Patients**: Monitor the health of staff who were in contact with the patient and ensure that they are aware of any symptoms of COVID-19. 8. **Reporting and Documentation**: Document the incident and report it as required by your local health authority guidelines.  It's important to consult and follow the latest guidelines from health authorities like the CDC or WHO, as recommendations can change based on evolving knowledge about the virus. | | When dealing with a situation where a patient with COVID-19 underwent an aerosol-generating procedure in a clinic room, it's crucial to follow the guidelines set by the United States Centers for Disease Control and Prevention (CDC) for infection control and room cleaning. As of my last update in April 2023, the recommendations include: Room Closure and Ventilation: After an aerosol-generating procedure, it's recommended to keep the room closed for a certain period to allow enough air changes to remove potentially infectious particles. The duration depends on the air exchange rate in the room. Cleaning and Disinfection: Following the necessary waiting period, the room should be thoroughly cleaned and disinfected. This includes all surfaces and equipment in the room. Use EPA-registered disinfectants that are effective against SARS-CoV-2. Personal Protective Equipment (PPE): Staff involved in cleaning should wear appropriate PPE, which may include gloves, gowns, and eye protection, in addition to a respirator or facemask. Limiting Room Access: Access to the room should be restricted during the waiting period and until cleaning and disinfection are completed. Waste Disposal: Any waste generated from the patient or the procedure should be handled as medical waste and disposed of in accordance with local regulations. Monitoring and Training of Staff: Ensure that staff are trained on these procedures and understand the importance of adherence to these guidelines.  Please consult the most recent CDC guidelines or contact them directly for the most current recommendations, as guidelines can change based on new information and research regarding COVID-19. | | According to the CDC and other sources, here are the steps you should follow:  Room Vacancy: After an aerosol-generating procedure (AGP), the room should remain vacant for a certain period to allow air exchanges. The time depends on the type of room:  Regular room (6 air exchanges per hour): 60 minutes. Airborne isolation room/negative pressure room (at least 12 air exchanges per hour): 30 minutes. The time starts when the AGP is completed. Cleaning and Disinfection: Routine cleaning and disinfection procedures are appropriate for COVID-19 in healthcare settings. This includes applying an EPA-registered, hospital-grade disinfectant to frequently touched surfaces or objects for appropriate contact times as indicated on the product’s label.  PPE and Waste Disposal: Follow standard operating procedures for the containment and disposal of used PPE and regulated medical waste.  Linen: Follow standard operating procedures for containing and laundering used linen. Avoid shaking the linen.  Please note that these guidelines are subject to change as new information about COVID-19 becomes available, so it’s important to stay updated with the latest recommendations from health authorities. Also, local regulations may apply. If you’re unsure, please consult with your local health department or infection control team. Stay safe! | | According to the CDC and other sources, here are the steps you should follow after an aerosol-generating procedure (AGP) has been performed in a room with a COVID-19 patient:  Room Vacancy: After the AGP is completed, the room should remain vacant for a certain period of time. This time is based on air exchanges for that location. For a regular room (6 air exchanges per hour), the room should remain vacant for 60 minutes. For an airborne isolation room/negative pressure room (at least 12 air exchanges per hour), the room should remain vacant for 30 minutes.  Infection prevention and control Infection Control Measures: Reinforce basic infection control practices in healthcare facilities and among healthcare personnel. This includes strict adherence to and proper use of standard infection control measures, especially hand hygiene.  Personal protective equipment Personal Protective Equipment (PPE): Healthcare personnel should wear an N95 respirator or PAPR, face shield, gloves, and gown for the entirety of the AGP. Patient Care: Patients with known or suspected COVID-19 should be cared for in a single-person room with the door closed. Please note that these are general guidelines and may vary based on specific circumstances. Always refer to the most recent guidelines from the CDC and local health authorities. | |
|  | Accuracy: 2 | Completeness: 3 | Accuracy:3 | Completeness: 5 | Accuracy: 5 | Completeness: 5 | Accuracy:4 | Completeness: 6 | Accuracy: 3 | Completeness: 4 | Accuracy:4 | Completeness: 4 | Accuracy: 4 | Completeness: 4 | Accuracy:5 | Completeness: 6 |
